# Correction: Dichotomous role of the human mitochondrial Na^+^/Ca2^+^/Li^+^ exchanger NCLX in colorectal cancer growth and metastasis

**DOI:** 10.7554/eLife.86471

**Published:** 2023-02-09

**Authors:** Trayambak Pathak, Maxime Gueguinou, Vonn Walter, Celine Delierneux, Martin T Johnson, Xuexin Zhang, Ping Xin, Ryan E Yoast, Scott M Emrich, Gregory S Yochum, Israel Sekler, Walter A Koltun, Donald L Gill, Nadine Hempel, Mohamed Trebak

**Keywords:** Human, Mouse

Pathak T, Gueguinou M, Walter V, Delierneux C, Johnson MT, Zhang X, Xin P, Yoast RE, Emrich SM, Yochum GS, Sekler I, Koltun WA, Gill DL, Hempel N, Trebak M. YYYY. Dichotomous role of the human mitochondrial Na+/Ca2+/Li+ exchanger NCLX in colorectal cancer growth and metastasis. *eLife* **9**:e59686. doi: 10.7554/eLife.59686.

Published 11 September 2020

We have been made aware through PubPeer of a mistake in Figure 3—figure supplement 1. Although the issues identified on PubPeer do not change the conclusions of the paper, they warrant correction. All sequences of primers and guide RNAs used in generating knockout (KO) cell clones and in genotyping are correct. Unfortunately, two sets of sequences were inadvertently swapped by the first author, a confusion that was caused by the fact that the NCLX gene is reversed in mammals. Although there is no excuse for such errors, unfortunately none of the contributing authors noticed these issues. We corrected this and added the sequences of the primers for the housekeeping genes that were identified as missing. Therefore, we modified the Appendix 1—key resources table and the cartoon in Figure 3—figure supplement 1 to reflect these corrections. Finally, we provide a Supplementary file 1, which contains genomic sequencing data for all our NCLX KO clones that provide decisive evidence that the NCLX is indeed knocked out in these cells. The details of the corrections are as follow:

We have corrected the text in the Methods section to change the guide RNA from g1 to g2 and add the predicted location of the stop codon for NCLX KO #33. The corrected text is shown here:

For the case of HCT116 cells, which we generated first, NCLX KO #33 was generated using a guide RNA (g2) which resulted in a single cut at nucleotide 150 in exon one causing a frameshift mutation and introduction of a stop codon at predicted position 181–183 bp and 184–186 bp in the NCLX open reading frame (Figure 3—figure supplement 1A).

Original text for reference:

For the case of HCT116 cells, which we generated first, NCLX KO #33 was generated using a guide RNA (g1) which resulted in a single cut at nucleotide 150 in exon one causing a frameshift mutation and introduction of a stop codon at position 180 in the NCLX open reading frame (Figure 3—figure supplement 1A).

We have corrected the text in the Results section to change the guide RNA from g1 to g2 and add the predicted location of the stop codon for NCLX KO #33. The corrected text is shown here:

For in vivo studies NCLX KO clone #33 was used, which was generated using a guide RNA (g2) resulting in a single cut at nucleotide 150 in exon one causing a frameshift mutation and introduction of a stop codon at predicted position 181–183 bp and 184–186 bp in the NCLX open reading frame (Figure 2A).

Original text for reference:

For in vivo studies NCLX KO clone #33 was used, which was generated using a guide RNA (g1) resulting in a single cut at nucleotide 150 in exon one causing a frameshift mutation and introduction of a stop codon at position 180 in the NCLX open reading frame (Figure 2A).

We have corrected the text in the Results section to fix the gene name from Fox3 to Foxo3. The corrected text is shown here:

In agreement with these findings, we found that transcript levels of stem cell markers NANOG, Oct4, Sox2, and Foxo3, as well as regulators of the glutathione synthesis pathway implicated in regulating these transcription factors in breast cancer, SLC7A11 and GCLM (Lu et al., 2015), were significantly upregulated in NCLX KO cells (Figure 5H–J, Figure 5—figure supplement 1G–O).

Original text for reference:

In agreement with these findings, we found that transcript levels of stem cell markers NANOG, Oct4, Sox2, and Fox3, as well as regulators of the glutathione synthesis pathway implicated in regulating these transcription factors in breast cancer, SLC7A11 and GCLM (Lu et al., 2015), were significantly upregulated in NCLX KO cells (Figure 5H–J, Figure 5—figure supplement 1G–O).

We have corrected the figure legend of Figure 5—figure supplement 1M to fix the gene name from Fox3 to Foxo3. The corrected text is shown here:

(G–N) RT-qPCR data plotted as the 2-ΔCt mRNA value of SLC7A11 (G), GCLM (H) in clones of HCT116 NCLX KO cells normalized to control HCT116 cells, and SLC7A11 (I), GCLM (J) NANOG (K), Sox2 (L), Foxo3 (M), and Oct4 (N) in clones of DLD1 NCLX KO cells relative to tubulin and normalized to control DLD1 cells.

Original text for reference:

(G–N) RT-qPCR data plotted as the 2-ΔCt mRNA value of SLC7A11 (G), GCLM (H) in clones of HCT116 NCLX KO cells normalized to control HCT116 cells, and SLC7A11 (I), GCLM (J) NANOG (K), Sox2 (L), Fox3 (M), and Oct4 (N) in clones of DLD1 NCLX KO cells relative to tubulin and normalized to control DLD1 cells.

The unfortunate confusion described above led to mislabelling of primers and the two guide RNA in the cartoon of Figure 3—figure supplement 1A-F. This confusion occurred because the NCLX gene is reversed in mammals. We now include an updated version of Figure 3—figure supplement 1, where the guide RNAs and primers within the cartoons are drawn as much to scale as possible with the relative positions of primers and guide RNAs and the predicted positions of the STOP codons introduced by CRISPR/Cas9. In the case of DLD1 cells in Figure 3—figure supplement 1G, the correct primer combination is 4+5 and not 3+6 as originally stated. The corrected Figure 3—figure supplement 1 is shown here:

**Figure 1:**
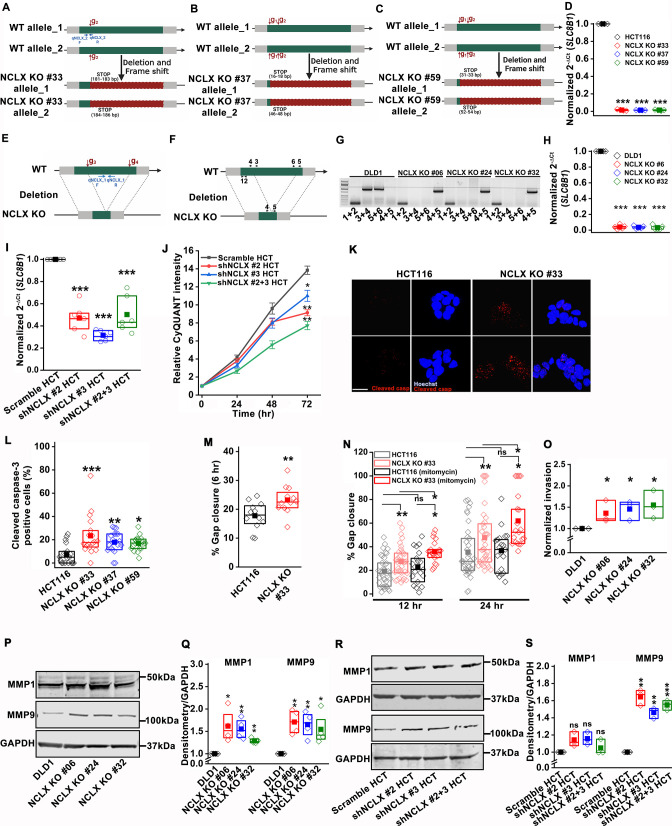


The originally published Figure 3—figure supplement 1 is shown for reference:

**Figure 2:**
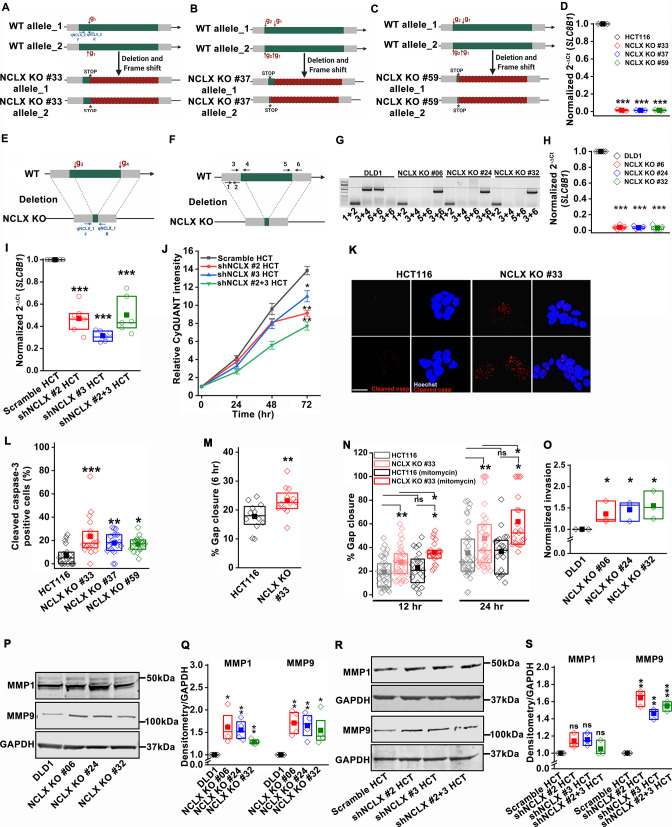


The original Figure 3—figure supplement 1A was reproduced in Figure 2A for convenience. Therefore, we also need to fix the cartoon in Figure 2A. The corrected Figure 2 is shown here:

**Figure 3:**
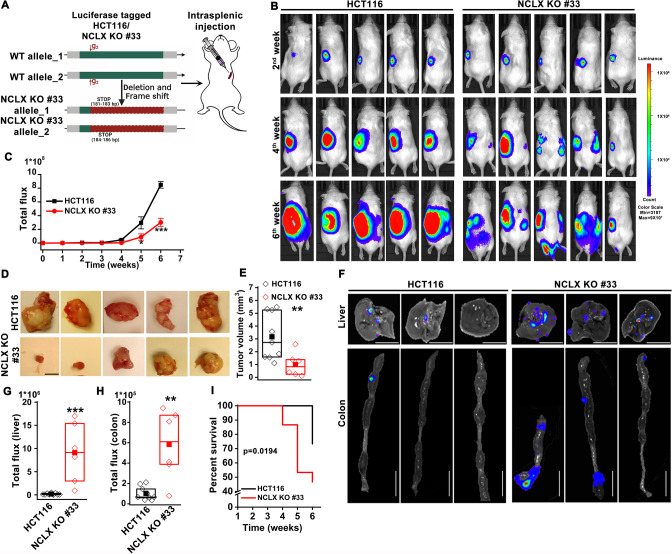


The originally published Figure 2 is shown for reference:

**Figure 4:**
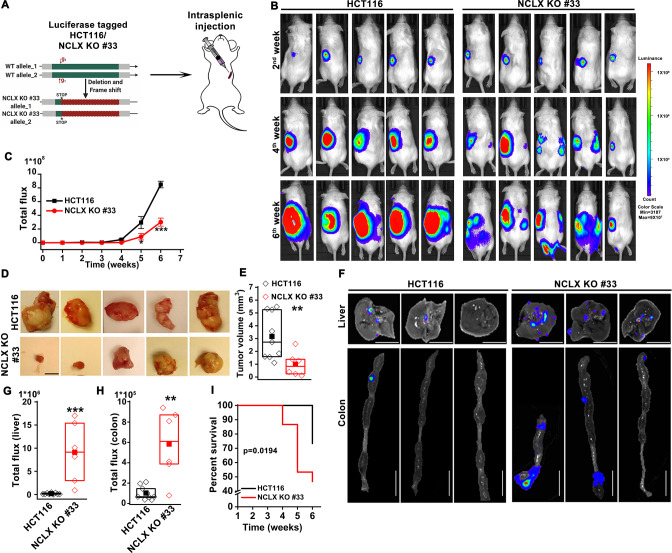


We also corrected the gene name Fox3 to Foxo3 in Figure 5—figure supplement 1M. The corrected Figure 5—figure supplement 1 is shown here:

**Figure 5:**
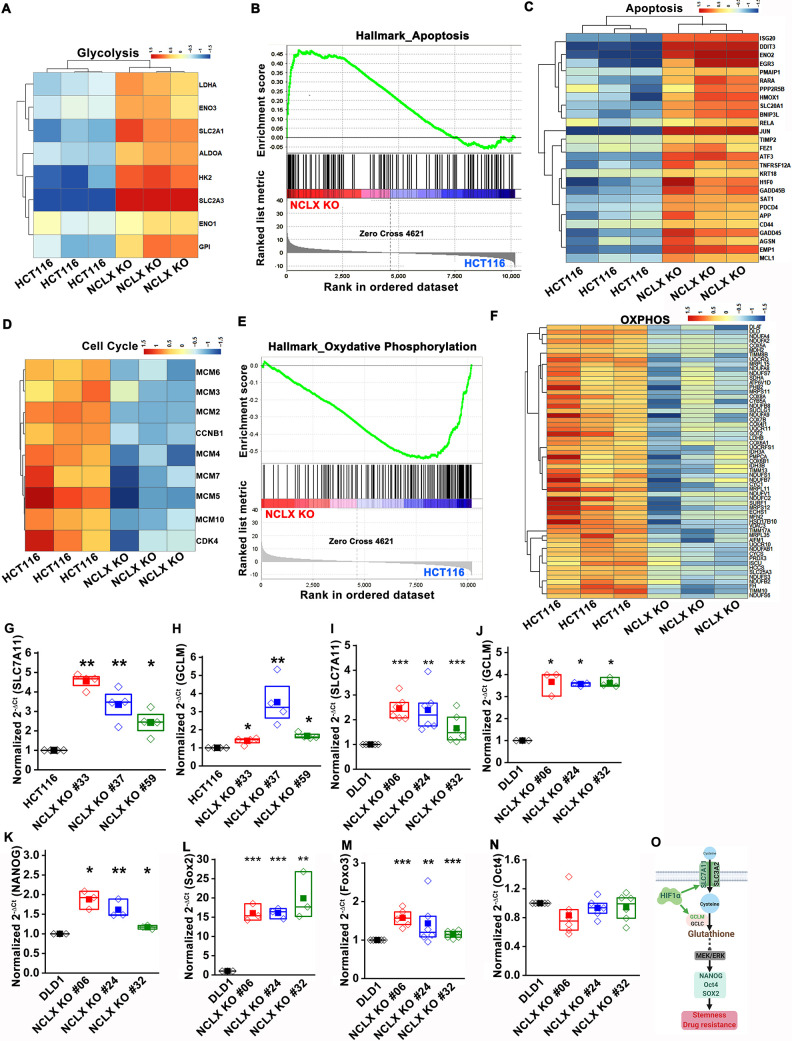


The original Figure 5—figure supplement 1 is shown here for reference:

**Figure 6:**
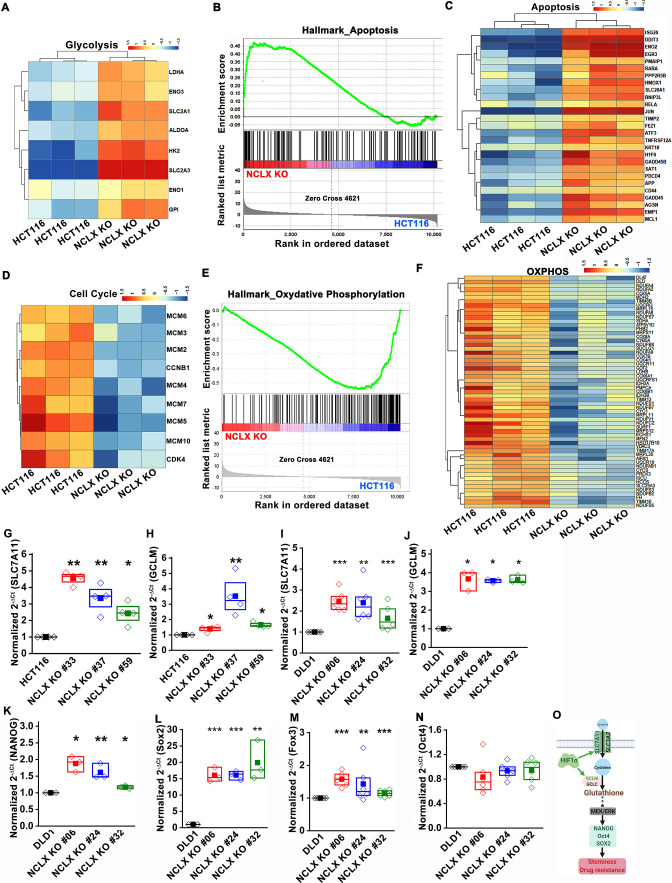


The GAPDH and FOXO3 primers were identified wrong, and the primers for two housekeeping genes, NONO and Tubulin, were missing from the Appendix 1—key resources table. We have updated the Appendix 1—key resources table to include these primer pairs. In addition, we have corrected the primer sequences of qNCLX1_F, qNCLX1_R, NCLX_1, and NCLX_2, which were inadvertently swapped in the original Appendix 1—key resources table. We have also corrected the name from HCT116 shNCLX KO and DLD1 shNCLX KO to HCT116 shNCLX and DLD1 shNCLX in the Appendix 1—key resources table. The latter mistakes happened because we uploaded the wrong version of the Appendix 1—key resources table in the original manuscript. The corrected Appendix 1—key resources table is shown here (The corrections are highlighted in bold for illustrative purposes):

Appendix 1—key resources table

**Table:** 

AReagent type(species) or resource	Designation	Source or reference	Identifiers	Additional information
Gene (*Homo sapiens*)	SLC8B1/NCLX	GenBank	Gene ID: 80024	
Gene *Mus musculus*	Slc8b1/NCLX	GenBank	Gene ID: 170756	
Genetic reagent *Mus musculus*	NOD.CB17-Prkdcscid/J	Jackson Laboratory	Stock No: 010636, RRID:IMSR_JAX:010636	Male
Cell line (Homo-sapiens)	HCT116 (colon, epithelial)	ATCC	ATCC# CCL-247	Male
Cell line (Homo-sapiens)	HT29 (colon, epithelial)	ATCC	ATCC# HTB-38	Female
Cell line (Homo-sapiens)	DLD1 (colon, epithelial)	ATCC	ATCC# CCL-221	Male
Cell line (Homo-sapiens)	HCT116 NCLX KO (colon, epithelial)	This paper		NCLX Knockout clones of HCT116 cells were generated by the Trebak lab using CRISPR/Cas9 and are available upon request
Cell line (Homo-sapiens)	DLD1 NCLX KO (colon, epithelial)	This paper		NCLX Knockout clones of DLD1 cells were generated in the Trebak lab using CRISPR/Cas9 and are available upon request
Cell line (Homo-sapiens)	HCT116 **shNCLX** (colon, epithelial)	This paper		HCT116 cells with stable shRNA-mediated knockdown of NCLX (shNCLX) were generated by the Trebak Lab using shRNA sequences (listed below in this table) cloned in the lentiviral vector pLKO. These plasmids are available upon request
Cell line (Homo-sapiens)	DLD1 **shNCLX** (colon, epithelial)	This paper		DLD1 cells with stable shRNA-mediated knockdown of NCLX (shNCLX) were generated by the Trebak Lab using shRNA sequences (listed below in this table) cloned in the lentiviral vector pLKO. These plasmids are available upon request
Antibody	anti-Hif1α (Rabbit polyclonal)	Cell Signaling Technology	Cat# 14,179 s, RRID:AB_2622225	WB (1:500)
Antibody	anti- ALDOA (Rabbit polyclonal)	Cell Signaling Technology	Cat# 8060 S, RRID:AB_2797635	WB (1:1000)
Antibody	anti- HK2 (Rabbit polyclonal)	Cell Signaling Technology	Cat# 2867 S, RRID:AB_2232946	WB (1:1000)
Antibody	anti- LDHA (Rabbit polyclonal)	Cell Signaling Technology	Cat# 2012 S, RRID:AB_2137173	WB (1:1000)
Antibody	anti- MMP1 (Rabbit polyclonal)	Abcam	Cat# ab38929, RRID:AB_776395	WB (1:1000)
Antibody	anti- MMP2 (Mouse monoclonal)	Santa Cruz Biotechnology	Cat# sc-13594, RRID:AB_627956	WB (1:500)
Antibody	anti- MMP9 (Rabbit polyclonal)	Abcam	Cat# ab73734, RRID:AB_1860201	WB (1:1000)
Antibody	anti- LC3B (Rabbit polyclonal)	Abcam	Cat# ab51520, RRID:AB_881429	WB (1:1000)
Antibody	anti- OXPHOS (Mouse monoclonal)	Abcam	Cat# ab110413, RRID:AB_2629281	WB (1:5000)
Antibody	anti- GAPDH (Mouse monoclonal)	Millipore Sigma	Cat# MAB374, RRID:AB_2107445	WB (1:10000)
Antibody	anti- HSC70 (Mouse monoclonal)	Santa Cruz Biotechnology	Cat# sc-24, RRID:AB_627760	WB (1:5000)
Antibody	anti- p62 (Rabbit polyclonal)	Abcam	Cat# ab109012, RRID:AB_2810880	WB (1:1000)
Antibody	anti- cleaved caspase-3 (Rabbit polyclonal)	Cell Signaling Technology	Cat# 9661 S, RRID:AB_2341188	WB (1:1000), IF (1:500)
Antibody	anti- pAMPK (Rabbit polyclonal)	Cell Signaling Technology	Cat# 2535 S, RRID:AB_331250	WB (1:1000)
Antibody	anti- AMPK (Rabbit polyclonal)	Cell Signaling Technology	Cat# 5831 S, RRID:AB_10622186	WB (1:1000)
Antibody	anti- pS6K (Rabbit polyclonal)	Cell Signaling Technology	Cat# 9234 S, RRID:AB_2269803	WB (1:1000)
Antibody	anti- S6K (Rabbit polyclonal)	Cell Signaling Technology	Cat# 2708 S, RRID:AB_390722	WB (1:1000)
Sequence-based reagent	SLC7A11_F	This paper	RT-PCR primers	AGGGTCACCTTCCAGAAATC
Sequence-based reagent	SLC7A11_R	This paper	RT-PCR primers	GAAGATAAATCAGCCCAGCA
Sequence-based reagent	GCLM _F	This paper	RT-PCR primers	CATTTACAGCCTTACTGGGAGG
Sequence-based reagent	GCLM _R	This paper	RT-PCR primers	ATGCAGTCAAATCTGGTGGCA
**Sequence-based reagent**	**FOXO3_F**	**This paper**	**RT-PCR primers**	**CGGACAAACGGCTCACTCT**
**Sequence-based reagent**	**FOXO3_R**	**This paper**	**RT-PCR primers**	**GGACCCGCATGAATCGACTAT**
Sequence-based reagent	NANOG _F	This paper	RT-PCR primers	TTTGTGGGCCTGAAGAAAACT
Sequence-based reagent	NANOG _R	This paper	RT-PCR primers	AGGGCTGTCCTGAATAAGCAG
Sequence-based reagent	OCT4_F	This paper	RT-PCR primers	TTCAGCCAAACGACCATCTG
Sequence-based reagent	OCT4_R	This paper	RT-PCR primers	CACGAGGGTTTCTGCTTTGC
Sequence-based reagent	SOX2_F	This paper	RT-PCR primers	GCCGAGTGGAAACTTTTGTCG
Sequence-based reagent	SOX2_R	This paper	RT-PCR primers	GGCAGCGTGTACTTATCCTTCT
Sequence-based reagent	GLUT1_F	This paper	RT-PCR primers	TATCGTCAACACGGCCTTCACT
Sequence-based reagent	GLUT1_R	This paper	RT-PCR primers	AACAGCTCCTCGGGTGTCTTAT
Sequence-based reagent	HK2_F	This paper	RT-PCR primers	GCCATCCTGCAACACTTAGGG
Sequence-based reagent	HK2_R	This paper	RT-PCR primers	GTGAGGATGTAGCTTGTAGAGGGT
Sequence-based reagent	GPI_F	This paper	RT-PCR primers	TGTGTTCACCAAGCTCACAC
Sequence-based reagent	GPI_R	This paper	RT-PCR primers	GTAGAAGCGTCGTGAGAGGT
Sequence-based reagent	ALDOA_F	This paper	RT-PCR primers	AGGCCATGCTTGCACTCAG
Sequence-based reagent	ALDOA_R	This paper	RT-PCR primers	AGGGCCCAGGGCTTCAG
Sequence-based reagent	ENO1_F	This paper	RT-PCR primers	GACTTGGCTGGCAACTCTG
Sequence-based reagent	ENO1_R	This paper	RT-PCR primers	GGTCATCGGGAGACTTGAAG
Sequence-based reagent	LDHA_F	This paper	RT-PCR primers	GGTTGGTGCTGTTGGCATGG
Sequence-based reagent	LDHA_R	This paper	RT-PCR primers	TGCCCCAGCCGTGATAATGA
Sequence-based reagent	MMP1_F	This paper	RT-PCR primers	ATGCTGAAACCCTGAAGGTG
Sequence-based reagent	MMP1_R	This paper	RT-PCR primers	GAGCATCCCCTCCAATACCT
Sequence-based reagent	MMP2_F	This paper	RT-PCR primers	ACCAGCTGGCCTAGTGATGATG
Sequence-based reagent	MMP2_R	This paper	RT-PCR primers	GGCTTCCGCATGGTCTCGATG
Sequence-based reagent	MMP9_F	This paper	RT-PCR primers	ACGCACGACGTCTTCCAGTA
Sequence-based reagent	MMP9_R	This paper	RT-PCR primers	CCACCTGGTTCAACTCACTCC
**Sequence-based reagent**	**qNCLX_1_F**	**This paper**	**RT-PCR primers**	**GCGTGCTGGTTACCACAGT**
**Sequence-based reagent**	**qNCLX_1_R**	**This paper**	**RT-PCR primers**	**CCACGGAAGAGCATGAGGAA**
Sequence-based reagent	qNCLX_2_F	This paper	RT-PCR primers	CCGGCAGAAGGCTGAATCTG
Sequence-based reagent	qNCLX_2_R	This paper	RT-PCR primers	ACCTTGCGGCAGTCTACCAC
**Sequence-based reagent**	**GAPDH_F**	**This paper**	**RT-PCR primers**	**CCCTTCATTGACCTCAACTACA**
**Sequence-based reagent**	**GAPDH_R**	**This paper**	**RT-PCR primers**	**ATGACAAGCTTCCCGTTCTC**
**Sequence-based reagent**	**NONO_F**	**This paper**	**RT-PCR primers**	**TCCGAGGAGATACCAGTCGG**
**Sequence-based reagent**	**NONO_R**	**This paper**	**RT-PCR primers**	**CCTGGGCCTCTCAACTTCGAT**
**Sequence-based reagent**	**Tubulin_F**	**This paper**	**RT-PCR primers**	**AGTCCAAGCTGGAGTTCTCTAT**
**Sequence-based reagent**	**Tubulin_R**	**This paper**	**RT-PCR primers**	**CAATCAGAGTGCTCCAGGGT**
Sequence-based reagent	G6PD_F	This paper	RT-PCR primers	CGAGGCCGTCACCAAGAAC
Sequence-based reagent	G6PD_R	This paper	RT-PCR primers	GTAGTGGTCGATGCGGTAGA
Sequence-based reagent	PGD_F	This paper	RT-PCR primers	ATGGCCCAAGCTGACATCG
Sequence-based reagent	PGD_R	This paper	RT-PCR primers	AAAGCCGTGGTCATTCATGTT
Sequence-based reagent	TKT_F	This paper	RT-PCR primers	TCCACACCATGCGCTACAAG
Sequence-based reagent	TKT_R	This paper	RT-PCR primers	CAAGTCGGAGCTGATCTTCCT
Sequence-based reagent	g1	This paper	Guide RNA sequences (Figure 2A and Figure 3—figure supplement 1A)	GCGCAGATTCAGCCTTCTGC
Sequence-based reagent	g2	This paper	Guide RNA sequences (Figure 3—figure supplement 1B and C)	GGGATACTCACGTCTACCAC
Sequence-based reagent	g3	This paper	Guide RNA sequences (Figure 3—figure supplement 1E)	GTAGACGTGAGTATCCCGGT
Sequence-based reagent	g4	This paper	Guide RNA sequences (Figure 3—figure supplement 1E)	ACCCACACCAGCAGTCCGTC
Sequence-based reagent	shRNA (shNCLX#2)	This paper	Figure 3—figure supplement 1I	GCCTTCTTGCTGTCATGCAAT
Sequence-based reagent	shRNA (shNCLX#3)	This paper	Figure 3—figure supplement 1I	GCTCCTCTTCTACCTGAACTT
Sequence-based reagent	siRNA (siNCLX)	This paper	Figure 4—figure supplement 1M	AACGGCCCCUCAACUGUCUT
**Sequence-based reagent**	**NCLX_1**	**This paper**	**PCR primers for screening genomic DNA of NCLX KO clones (Figure 3—figure supplement 1F**)	**GCCAGCATTTGTGTCCATTT**
**Sequence-based reagent**	**NCLX_2**	**This paper**	**PCR primers for screening genomic DNA of NCLX KO clones (Figure 3—figure supplement 1F**)	**AATTCGTCTCGGCCACTTAC**
Sequence-based reagent	NCLX_3	This paper	PCR primers for screening genomic DNA of NCLX KO clones (Figure 3—figure supplement 1F)	ACTTAGCACATCGCCACCTG
Sequence-based reagent	NCLX_4	This paper	PCR primers for screening genomic DNA of NCLX KO clones (Figure 3—figure supplement 1F)	CTGATCTGCACGCTGAATGG
Sequence-based reagent	NCLX_5	This paper	PCR primers for screening genomic DNA of NCLX KO clones (Figure 3—figure supplement 1F)	GAGGTACACAGCAGTTCT CCC
Sequence-based reagent	NCLX_6	This paper	PCR primers for screening genomic DNA of NCLX KO clones (Figure 3—figure supplement 1F)	CAGCTGGTGCCCTCAAACAC
**Sequence-based reagent**	**PX75 NCLX test F**	**This paper**	**PCR primers for screening genomic DNA of NCLX KO HCT116 cells**	**GTTGTTGAGACAGAGTCTTGC TTC**
**Sequence-based reagent**	**PX76 NCLX test R**	**This paper**	**PCR primers for screening genomic DNA of NCLX KO HCT116 cells**	**TCCAGCGAGACTGTGCAGAA**
Sequence-based reagent	px77	This paper	PCR primers for genotyping NCLX -/- mice	TACAGTCTGGCTCGTTCC CT
Sequence-based reagent	px78	This paper	PCR primers for genotyping NCLX -/- mice	CGGTCCCAGACGCCG T
Sequence-based reagent	px79	This paper	PCR primers for genotyping NCLX -/- mice	CGCTGGGGTCCATCT TTG AT
Sequence-based reagent	px80	This paper	PCR primers for genotyping NCLX -/- mice	TGGGTCTCCGGTCCCAGT A
Commercial assay or kit	cDNA Reverse Transcription Kit	Applied biosystems	Cat# 4368814	
Commercial assay or kit	Seahorse XFp Mito Fuel Flex Test Kit	Agilent Technologies	Cat# 103270–100	
Commercial assay or kit	Seahorse XFp Glycolysis Stress Test Kit	Agilent Technologies	Cat# 103017–100	
Commercial assay or kit	Seahorse XFp Cell Mito Stress Test Kit	Agilent Technologies	Cat# 103010–100	
Commercial assay or kit	BCA assay kit	Thermo Fisher Scientific	Cat# A53225	
Commercial assay or kit	TMRE	Thermo Fisher Scientific	Cat# T669	
Commercial assay or kit	CyQUANT	Thermo Fisher Scientific	Cat# C35006	
Chemical compound, drug	Antibiotic and Antimycotic	Thermo Fisher Scientific	Cat# 15240062	
Chemical compound, drug	McCoy’s 5 A	Corning	Cat# 10-050CV	
Chemical compound, drug	RPMI-1640	Corning	Cat# 10-040CV	
Chemical compound, drug	Lipofectamine 2000	Thermo Fisher Scientific	Cat# 11668019	
Chemical compound, drug	TrypLE	Thermo Fisher Scientific	Cat# 12605028	
Chemical compound, drug	CoCl_2_	Sigma-Aldrich	Cat# 15862	
Chemical compound, drug	2-deoxy-D-glucose (2-DG)	Sigma-Aldrich	Cat# D8375	
Chemical compound, drug	5-Fluorouracil	Sigma-Aldrich	Cat# F6627	
Chemical compound, drug	Glucose	Sigma-Aldrich	Cat# D9434	
Chemical compound, drug	Puromycin	MP Biomedical	Cat# 02100552	
Chemical compound, drug	RIPA buffer	Sigma	Cat# R0278	
Chemical compound, drug	ATP	Sigma	Cat# A9187	
Chemical compound, drug	Dextrose	Fisher Scientific	Cat# D14	
Chemical compound, drug	Tris Base	Fisher Scientific	Cat# BP152-5	
Chemical compound, drug	NaCl	Fisher Scientific	Cat# S671	
Chemical compound, drug	MOPS SDS running buffer	Thermo Fisher Scientific	Cat# NP0001	
Chemical compound, drug	Tris-Glycine transfer buffer	Bio-rad	Cat#161–0734	
Chemical compound, drug	KCl	Fisher Scientific	Cat# P217	
Chemical compound, drug	MgCl_2_	Fisher Scientific	Cat# M33	
Chemical compound, drug	CaCl_2_	Fisher Scientific	Cat# C614	
Chemical compound, drug	HEPES	Fisher Scientific	Cat# BP310	
Chemical compound, drug	LDS sample buffer	Thermo Fisher Scientific	Cat# NP0007	
Chemical compound, drug	NuPAGE Bis-Tris precast gels	Thermo Fisher Scientific	Cat# NP0321	
Chemical compound, drug	Polyvinylidene difluoride membrane	Li-Core Biosciences	Cat# 88518	
Chemical compound, drug	Odyssey Blocking Buffer (TBS)	Li-Core Biosciences	Cat# 937–50003	
Chemical compound, drug	Dextran sulfate sodium	MP Biomedical	Cat# 0216011080	
Chemical compound, drug	Azoxymethane	Sigma	Cat# A5486	
Chemical compound, drug	DNase I	Thermo Fisher Scientific	Cat# 18068–015	
Chemical compound, drug	TRIzol	Thermo Fisher Scientific	Cat# 15596018	
Chemical compound, drug	Seahorse XF DMEM Medium pH 7.4	Agilent Technologies	Cat# 103575–100	
Chemical compound, drug	Seahorse XF 100 mM pyruvate solution	Agilent Technologies	Cat# 103578–100	
Chemical compound, drug	Seahorse XF 200 mM glutamine solution	Agilent Technologies	Cat# 103579–100	
Chemical compound, drug	Seahorse XF 1.0 M glucose solution	Agilent Technologies	Cat# 103577–100	
Chemical compound, drug	Zymogram Developing Buffer (10 X)	Thermo Fisher Scientific	Cat# LC2671	
Chemical compound, drug	Zymogram Renaturing Buffer (10 X)	Thermo Fisher Scientific	Cat# LC2670	
Chemical compound, drug	Tris-Glycine SDS Running Buffer (10 X)	Thermo Fisher Scientific	Cat# LC2675	
Chemical compound, drug	Tween 20	Fisher Scientific	Cat# BP337	
Software, algorithm	Image J	https://imagej.net/	RRID:SCR_003070	
Other	DAPI	Sigma-Aldrich	Cat# D9542	1 µg/ml
Other	Hoechst	Thermo Fisher Scientific	Cat# H3570	1 µg/ml
Other	IRDye 800CW Goat anti-Mouse	Li-Core Biosciences	Cat# 925–32210	1:10000
Other	IRDye 800CW Donkey anti-Rabbit	Li-Core Biosciences	Cat# 925–32213	1:5000
Other	MitoSox Red	Thermo Fisher Scientific	Cat# M36008	
Other	Mito TEMPO	Thermo Fisher Scientific	Cat# SML0737	
Other	Mito Tracker Green FM	Thermo Fisher Scientific	Cat# M7514	
Other	Mito Tracker Deep red FM	Cell Signaling Technology	Cat# 8778 S	
Other	Fura-2 AM	Thermo Fisher Scientific	Cat# F1221	
Other	FluoroBlok	Corning	Cat# 351152	
Other	BioCoat Tumor Invasion Plate	Corning	Cat# 80774380	
Other	SYBER select master mix	Thermo Fisher Scientific	Cat# 4472920	
Other	Novex 10% Zymogram Plus (Gelatin) Protein Gels, 1.0 mm, 10-well	Thermo Fisher Scientific	Cat# ZY00100	
Other	SimplyBlue Safe Stain	Thermo Fisher Scientific	Cat# LC6060	

The originally published Appendix 1—key resources table is shown for reference:

Appendix 1—key resources table

**Table:** 

Reagent type(species) or resource	Designation	Source or reference	Identifiers	Additional information
Gene (*Homo sapiens*)	SLC8B1/NCLX	GenBank	Gene ID: 80024	
Gene *Mus musculus*	Slc8b1/NCLX	GenBank	Gene ID: 170756	
Genetic reagent *Mus musculus*	NOD.CB17-Prkdcscid/J	Jackson Laboratory	Stock No: 010636, RRID:IMSR_JAX:010636	Male
Cell line (Homo-sapiens)	HCT116 (colon, epithelial)	ATCC	ATCC# CCL-247	Male
Cell line (Homo-sapiens)	HT29 (colon, epithelial)	ATCC	ATCC# HTB-38	Female
Cell line (Homo-sapiens)	DLD1 (colon, epithelial)	ATCC	ATCC# CCL-221	Male
Cell line (Homo-sapiens)	HCT116 NCLX KO (colon, epithelial)	This paper		NCLX Knockout clones of HCT116 cells were generated by the Trebak lab using CRISPR/Cas9 and are available upon request
Cell line (Homo-sapiens)	DLD1 NCLX KO (colon, epithelial)	This paper		NCLX Knockout clones of DLD1 cells were generated in the Trebak lab using CRISPR/Cas9 and are available upon request
Cell line (Homo-sapiens)	HCT116 shNCLX KO (colon, epithelial)	This paper		HCT116 cells with stable shRNA-mediated knockdown of NCLX (shNCLX) were generated by the Trebak Lab using shRNA sequences (listed below in this table) cloned in the lentiviral vector pLKO. These plasmids are available upon request
Cell line (Homo-sapiens)	DLD1 shNCLX KO (colon, epithelial)	This paper		DLD1 cells with stable shRNA-mediated knockdown of NCLX (shNCLX) were generated by the Trebak Lab using shRNA sequences (listed below in this table) cloned in the lentiviral vector pLKO. These plasmids are available upon request
Antibody	anti-Hif1α (Rabbit polyclonal)	Cell Signaling Technology	Cat# 14,179 s, RRID:AB_2622225	WB (1:500)
Antibody	anti- ALDOA (Rabbit polyclonal)	Cell Signaling Technology	Cat# 8060 S, RRID:AB_2797635	WB (1:1000)
Antibody	anti- HK2 (Rabbit polyclonal)	Cell Signaling Technology	Cat# 2867 S, RRID:AB_2232946	WB (1:1000)
Antibody	anti- LDHA (Rabbit polyclonal)	Cell Signaling Technology	Cat# 2012 S, RRID:AB_2137173	WB (1:1000)
Antibody	anti- MMP1 (Rabbit polyclonal)	Abcam	Cat# ab38929, RRID:AB_776395	WB (1:1000)
Antibody	anti- MMP2 (Mouse monoclonal)	Santa Cruz Biotechnology	Cat# sc-13594, RRID:AB_627956	WB (1:500)
Antibody	anti- MMP9 (Rabbit polyclonal)	Abcam	Cat# ab73734, RRID:AB_1860201	WB (1:1000)
Antibody	anti- LC3B (Rabbit polyclonal)	Abcam	Cat# ab51520, RRID:AB_881429	WB (1:1000)
Antibody	anti- OXPHOS (Mouse monoclonal)	Abcam	Cat# ab110413, RRID:AB_2629281	WB (1:5000)
Antibody	anti- GAPDH (Mouse monoclonal)	Millipore Sigma	Cat# MAB374, RRID:AB_2107445	WB (1:10000)
Antibody	anti- HSC70 (Mouse monoclonal)	Santa Cruz Biotechnology	Cat# sc-24, RRID:AB_627760	WB (1:5000)
Antibody	anti- p62 (Rabbit polyclonal)	Abcam	Cat# ab109012, RRID:AB_2810880	WB (1:1000)
Antibody	anti- cleaved caspase-3 (Rabbit polyclonal)	Cell Signaling Technology	Cat# 9661 S, RRID:AB_2341188	WB (1:1000), IF (1:500)
Antibody	anti- pAMPK (Rabbit polyclonal)	Cell Signaling Technology	Cat# 2535 S, RRID:AB_331250	WB (1:1000)
Antibody	anti- AMPK (Rabbit polyclonal)	Cell Signaling Technology	Cat# 5831 S, RRID:AB_10622186	WB (1:1000)
Antibody	anti- pS6K (Rabbit polyclonal)	Cell Signaling Technology	Cat# 9234 S, RRID:AB_2269803	WB (1:1000)
Antibody	anti- S6K (Rabbit polyclonal)	Cell Signaling Technology	Cat# 2708 S, RRID:AB_390722	WB (1:1000)
Sequence-based reagent	SLC7A11_F	This paper	RT-PCR primers	AGGGTCACCTTCCAGAAATC
Sequence-based reagent	SLC7A11_R	This paper	RT-PCR primers	GAAGATAAATCAGCCCAGCA
Sequence-based reagent	GCLM _F	This paper	RT-PCR primers	CATTTACAGCCTTACTGGGAGG
Sequence-based reagent	GCLM _R	This paper	RT-PCR primers	ATGCAGTCAAATCTGGTGGCA
Sequence-based reagent	FOX3_F	This paper	RT-PCR primers	CGGCTTCGGCTCTTAGCAAA
Sequence-based reagent	FOX3_R	This paper	RT-PCR primers	CGGACAAACGGCTCACTCT
Sequence-based reagent	NANOG _F	This paper	RT-PCR primers	TTTGTGGGCCTGAAGAAAACT
Sequence-based reagent	NANOG _R	This paper	RT-PCR primers	AGGGCTGTCCTGAATAAGCAG
Sequence-based reagent	OCT4_F	This paper	RT-PCR primers	TTCAGCCAAACGACCATCTG
Sequence-based reagent	OCT4_R	This paper	RT-PCR primers	CACGAGGGTTTCTGCTTTGC
Sequence-based reagent	SOX2_F	This paper	RT-PCR primers	GCCGAGTGGAAACTTTTGTCG
Sequence-based reagent	SOX2_R	This paper	RT-PCR primers	GGCAGCGTGTACTTATCCTTCT
Sequence-based reagent	GLUT1_F	This paper	RT-PCR primers	TATCGTCAACACGGCCTTCACT
Sequence-based reagent	GLUT1_R	This paper	RT-PCR primers	AACAGCTCCTCGGGTGTCTTAT
Sequence-based reagent	HK2_F	This paper	RT-PCR primers	GCCATCCTGCAACACTTAGGG
Sequence-based reagent	HK2_R	This paper	RT-PCR primers	GTGAGGATGTAGCTTGTAGAGGGT
Sequence-based reagent	GPI_F	This paper	RT-PCR primers	TGTGTTCACCAAGCTCACAC
Sequence-based reagent	GPI_R	This paper	RT-PCR primers	GTAGAAGCGTCGTGAGAGGT
Sequence-based reagent	ALDOA_F	This paper	RT-PCR primers	AGGCCATGCTTGCACTCAG
Sequence-based reagent	ALDOA_R	This paper	RT-PCR primers	AGGGCCCAGGGCTTCAG
Sequence-based reagent	ENO1_F	This paper	RT-PCR primers	GACTTGGCTGGCAACTCTG
Sequence-based reagent	ENO1_R	This paper	RT-PCR primers	GGTCATCGGGAGACTTGAAG
Sequence-based reagent	LDHA_F	This paper	RT-PCR primers	GGTTGGTGCTGTTGGCATGG
Sequence-based reagent	LDHA_R	This paper	RT-PCR primers	TGCCCCAGCCGTGATAATGA
Sequence-based reagent	MMP1_F	This paper	RT-PCR primers	ATGCTGAAACCCTGAAGGTG
Sequence-based reagent	MMP1_R	This paper	RT-PCR primers	GAGCATCCCCTCCAATACCT
Sequence-based reagent	MMP2_F	This paper	RT-PCR primers	ACCAGCTGGCCTAGTGATGATG
Sequence-based reagent	MMP2_R	This paper	RT-PCR primers	GGCTTCCGCATGGTCTCGATG
Sequence-based reagent	MMP9_F	This paper	RT-PCR primers	ACGCACGACGTCTTCCAGTA
Sequence-based reagent	MMP9_R	This paper	RT-PCR primers	CCACCTGGTTCAACTCACTCC
Sequence-based reagent	qNCLX_1_F	This paper	RT-PCR primers	GCCAGCATTTGTGTCCATTT
Sequence-based reagent	qNCLX_1_R	This paper	RT-PCR primers	AATTCGTCTCGGCCACTTAC
Sequence-based reagent	qNCLX_2_F	This paper	RT-PCR primers	CCGGCAGAAGGCTGAATCTG
Sequence-based reagent	qNCLX_2_R	This paper	RT-PCR primers	ACCTTGCGGCAGTCTACCAC
Sequence-based reagent	GAPDH_F	This paper	RT-PCR primers	GGGAAACCCATCACCATCTT
Sequence-based reagent	GAPDH_R	This paper	RT-PCR primers	CCAGTAGACTCCACGACATACT
Sequence-based reagent	G6PD_F	This paper	RT-PCR primers	CGAGGCCGTCACCAAGAAC
Sequence-based reagent	G6PD_R	This paper	RT-PCR primers	GTAGTGGTCGATGCGGTAGA
Sequence-based reagent	PGD_F	This paper	RT-PCR primers	ATGGCCCAAGCTGACATCG
Sequence-based reagent	PGD_R	This paper	RT-PCR primers	AAAGCCGTGGTCATTCATGTT
Sequence-based reagent	TKT_F	This paper	RT-PCR primers	TCCACACCATGCGCTACAAG
Sequence-based reagent	TKT_R	This paper	RT-PCR primers	CAAGTCGGAGCTGATCTTCCT
Sequence-based reagent	g1	This paper	Guide RNA sequences (Figure 2A and Figure 3—figure supplement 1A)	GCGCAGATTCAGCCTTCTGC
Sequence-based reagent	g2	This paper	Guide RNA sequences (Figure 3—figure supplement 1B and C)	GGGATACTCACGTCTACCAC
Sequence-based reagent	g3	This paper	Guide RNA sequences (Figure 3—figure supplement 1E)	GTAGACGTGAGTATCCCGGT
Sequence-based reagent	g4	This paper	Guide RNA sequences (Figure 3—figure supplement 1E)	ACCCACACCAGCAGTCCGTC
Sequence-based reagent	shRNA (shNCLX#2)	This paper	Figure 3—figure supplement 1I	GCCTTCTTGCTGTCATGCAAT
Sequence-based reagent	shRNA (shNCLX#3)	This paper	Figure 3—figure supplement 1I	GCTCCTCTTCTACCTGAACTT
Sequence-based reagent	siRNA (siNCLX)	This paper	Figure 4—figure supplement 1M	AACGGCCCCUCAACUGUCUT
Sequence-based reagent	NCLX_1	This paper	PCR primers for screening genomic DNA of NCLX KO clones (Figure 3—figure supplement 1F)	GCGTGCTGGTTACCACAG T
Sequence-based reagent	NCLX_2	This paper	PCR primers for screening genomic DNA of NCLX KO clones (Figure 3—figure supplement 1F)	CCACGGAAGAGCATGAGGAA
Sequence-based reagent	NCLX_3	This paper	PCR primers for screening genomic DNA of NCLX KO clones (Figure 3—figure supplement 1F)	ACTTAGCACATCGCCACCTG
Sequence-based reagent	NCLX_4	This paper	PCR primers for screening genomic DNA of NCLX KO clones (Figure 3—figure supplement 1F)	CTGATCTGCACGCTGAATGG
Sequence-based reagent	NCLX_5	This paper	PCR primers for screening genomic DNA of NCLX KO clones (Figure 3—figure supplement 1F)	GAGGTACACAGCAGTTCT CCC
Sequence-based reagent	NCLX_6	This paper	PCR primers for screening genomic DNA of NCLX KO clones (Figure 3—figure supplement 1F)	CAGCTGGTGCCCTCAAACAC
Sequence-based reagent	px77	This paper	PCR primers for genotyping NCLX -/- mice	TACAGTCTGGCTCGTTCC CT
Sequence-based reagent	px78	This paper	PCR primers for genotyping NCLX -/- mice	CGGTCCCAGACGCCG T
Sequence-based reagent	px79	This paper	PCR primers for genotyping NCLX -/- mice	CGCTGGGGTCCATCT TTG AT
Sequence-based reagent	px80	This paper	PCR primers for genotyping NCLX -/- mice	TGGGTCTCCGGTCCCAGT A
Commercial assay or kit	cDNA Reverse Transcription Kit	Applied biosystems	Cat# 4368814	
Commercial assay or kit	Seahorse XFp Mito Fuel Flex Test Kit	Agilent Technologies	Cat# 103270–100	
Commercial assay or kit	Seahorse XFp Glycolysis Stress Test Kit	Agilent Technologies	Cat# 103017–100	
Commercial assay or kit	Seahorse XFp Cell Mito Stress Test Kit	Agilent Technologies	Cat# 103010–100	
Commercial assay or kit	BCA assay kit	Thermo Fisher Scientific	Cat# A53225	
Commercial assay or kit	TMRE	Thermo Fisher Scientific	Cat# T669	
Commercial assay or kit	CyQUANT	Thermo Fisher Scientific	Cat# C35006	
Chemical compound, drug	Antibiotic and Antimycotic	Thermo Fisher Scientific	Cat# 15240062	
Chemical compound, drug	McCoy’s 5 A	Corning	Cat# 10-050CV	
Chemical compound, drug	RPMI-1640	Corning	Cat# 10-040CV	
Chemical compound, drug	Lipofectamine 2000	Thermo Fisher Scientific	Cat# 11668019	
Chemical compound, drug	TrypLE	Thermo Fisher Scientific	Cat# 12605028	
Chemical compound, drug	CoCl_2_	Sigma-Aldrich	Cat# 15862	
Chemical compound, drug	2-deoxy-D-glucose (2-DG)	Sigma-Aldrich	Cat# D8375	
Chemical compound, drug	5-Fluorouracil	Sigma-Aldrich	Cat# F6627	
Chemical compound, drug	Glucose	Sigma-Aldrich	Cat# D9434	
Chemical compound, drug	Puromycin	MP Biomedical	Cat# 02100552	
Chemical compound, drug	RIPA buffer	Sigma	Cat# R0278	
Chemical compound, drug	ATP	Sigma	Cat# A9187	
Chemical compound, drug	Dextrose	Fisher Scientific	Cat# D14	
Chemical compound, drug	Tris Base	Fisher Scientific	Cat# BP152-5	
Chemical compound, drug	NaCl	Fisher Scientific	Cat# S671	
Chemical compound, drug	MOPS SDS running buffer	Thermo Fisher Scientific	Cat# NP0001	
Chemical compound, drug	Tris-Glycine transfer buffer	Bio-rad	Cat#161–0734	
Chemical compound, drug	KCl	Fisher Scientific	Cat# P217	
Chemical compound, drug	MgCl_2_	Fisher Scientific	Cat# M33	
Chemical compound, drug	CaCl_2_	Fisher Scientific	Cat# C614	
Chemical compound, drug	HEPES	Fisher Scientific	Cat# BP310	
Chemical compound, drug	LDS sample buffer	Thermo Fisher Scientific	Cat# NP0007	
Chemical compound, drug	NuPAGE Bis-Tris precast gels	Thermo Fisher Scientific	Cat# NP0321	
Chemical compound, drug	Polyvinylidene difluoride membrane	Li-Core Biosciences	Cat# 88518	
Chemical compound, drug	Odyssey Blocking Buffer (TBS)	Li-Core Biosciences	Cat# 937–50003	
Chemical compound, drug	Dextran sulfate sodium	MP Biomedical	Cat# 0216011080	
Chemical compound, drug	Azoxymethane	Sigma	Cat# A5486	
Chemical compound, drug	DNase I	Thermo Fisher Scientific	Cat# 18068–015	
Chemical compound, drug	TRIzol	Thermo Fisher Scientific	Cat# 15596018	
Chemical compound, drug	Seahorse XF DMEM Medium pH 7.4	Agilent Technologies	Cat# 103575–100	
Chemical compound, drug	Seahorse XF 100 mM pyruvate solution	Agilent Technologies	Cat# 103578–100	
Chemical compound, drug	Seahorse XF 200 mM glutamine solution	Agilent Technologies	Cat# 103579–100	
Chemical compound, drug	Seahorse XF 1.0 M glucose solution	Agilent Technologies	Cat# 103577–100	
Chemical compound, drug	Zymogram Developing Buffer (10 X)	Thermo Fisher Scientific	Cat# LC2671	
Chemical compound, drug	Zymogram Renaturing Buffer (10 X)	Thermo Fisher Scientific	Cat# LC2670	
Chemical compound, drug	Tris-Glycine SDS Running Buffer (10 X)	Thermo Fisher Scientific	Cat# LC2675	
Chemical compound, drug	Tween 20	Fisher Scientific	Cat# BP337	
Software, algorithm	Image J	https://imagej.net/	RRID:SCR_003070	
Other	DAPI	Sigma-Aldrich	Cat# D9542	1 µg/ml
Other	Hoechst	Thermo Fisher Scientific	Cat# H3570	1 µg/ml
Other	IRDye 800CW Goat anti-Mouse	Li-Core Biosciences	Cat# 925–32210	1:10000
Other	IRDye 800CW Donkey anti-Rabbit	Li-Core Biosciences	Cat# 925–32213	1:5000
Other	MitoSox Red	Thermo Fisher Scientific	Cat# M36008	
Other	Mito TEMPO	Thermo Fisher Scientific	Cat# SML0737	
Other	Mito Tracker Green FM	Thermo Fisher Scientific	Cat# M7514	
Other	Mito Tracker Deep red FM	Cell Signaling Technology	Cat# 8778 S	
Other	Fura-2 AM	Thermo Fisher Scientific	Cat# F1221	
Other	FluoroBlok	Corning	Cat# 351152	
Other	BioCoat Tumor Invasion Plate	Corning	Cat# 80774380	
Other	SYBER select master mix	Thermo Fisher Scientific	Cat# 4472920	
Other	Novex 10% Zymogram Plus (Gelatin) Protein Gels, 1.0 mm, 10-well	Thermo Fisher Scientific	Cat# ZY00100	
Other	SimplyBlue Safe Stain	Thermo Fisher Scientific	Cat# LC6060	

**Supplementary file 1: Genomic sequencing of NCLX KO clones of HCT116 and DLD1 cells.**

**Description:** This file shows the genome sequencing of NCLX KO clones #33, #37, and #59 of HCT116 and #06, #24, and #32 of DLD1 cells. For genomic sequencing, PCR was performed to amplify specific parts of the NCLX gene using primers listed in the key resources table. The primers used for cloning genomic DNA from HCT116 cells were PX75 NCLX test F and PX76 NCLX test R. The primers used for cloning genomic DNA from DLD1 cells were NCLX_4 and NCLX_5. PCR products were cloned using StrataClone Blunt PCR Cloning Kit and sent to Genewiz for sanger sequencing. The genome sequencing data for the HCT116 NCLX KO clones confirm the introduction of STOP codons in coding sequences of HCT116 NCLX KO #33 at predicted positions 181–183 bp and 184–186 bp, in HCT116 NCLX KO #37 at 16–18 bp and 46–48 bp, and in HCT116 NCLX KO #59 at 31–33 bp, and 52–54 bp. Furthermore, the genomic sequencing of NCLX KO clones of DLD1 cells showed that the middle ~32 kb portion of the NCLX gene was deleted in all clones. Therefore, providing decisive evidence that these cells are indeed NCLX KO.

The corrections do not change any of the scientific conclusions of the figures or the manuscript. We sincerely apologize for the mistakes and the confusion it may have caused.

The article has been corrected accordingly.

