# Supplementary material for: Dichotomous role of the human mitochondrial Na+/Ca2+/Li+ exchanger NCLX in colorectal cancer growth and metastasis
Source: eLife. 2020 Sep 11;9:e59686. doi: 10.7554/eLife.59686 (PMC7529464; doi:10.7554/eLife.59686)
Supplement: Supplementary file 1. — This file shows the genome sequencing of NCLX KO clones #33, #37, and #59 of HCT116 and #06, #24, and #32 of DLD1 cells. For genomic sequencing, PCR was performed to amplify specific parts of the NCLX gene using primers listed in the key resources table. The primers used for cloning genomic DNA from HCT116 cells were PX75 NCLX test F and PX76 NCLX test R. The primers used for cloning genomic DNA from DLD1 cells were NCLX_4 and NCLX_5. PCR products were cloned using StrataClone Blunt PCR Cloning Kit and sent to Genewiz for sanger sequencing. The genome sequencing data for the HCT116 NCLX KO clones confirm the introduction of STOP codons in coding sequences of HCT116 NCLX KO #33 at predicted positions 181-183 bp and 184-186 bp, in HCT116 NCLX KO #37 at 16-18 bp and 46-48 bp, and in HCT116 NCLX KO #59 at 31-33 bp, and 52-54 bp. Furthermore, the genomic sequencing of NCLX KO clones of DLD1 cells showed that the middle ~32 kb portion of the NCLX gene was deleted in all clones. Therefore, providing decisive evidence that these cells are indeed NCLX KO. [file elife-59686-supp1.docx]

**Supplementary file 1: Genomic sequencing of NCLX KO clones of HCT116 and DLD1 cells.**

**Sequencing of HCT116 NCLX KO Clones**

NCLX KO#33_1

NNNNNNNNNNTNNANGGCGATGGAGCTCCCGCGGTGCGGCCGCTCTAGAACTAGTGGATCCCCCGGGCTGCAGCGACCAATGTGGAATTCGCCCTTACTTAGCACATCGCCACCTGAAATGTATATGCTTACTTCTCTTTTTTTTGTTGTTGAGACAGAGTCTTGCTTCGCCGCCCAGGCTGGAGTGCAGTGGTACAATCCTGGCTCACTGCAACATCCACCTCACGGTTTCAAGCAATTCTCCTGCCTCAGGGTTCTGATGATTACAGGCACCTGCCACCATGCCCAGCTAATTTTTGTATTTTTAGTAGATACGGGGTTTTGACACGATGGCCAGGCTGGTCTCCAGCTCCTGGCCTCAAGTGATCCTCCTGCCTTGGCCTCCCAAAGTGTATAAGTTTACTTCTTGATCTGTCTGTTCAGGAGACTGTCAGCTTCTTGAGAGTAGTGAATTTTGTCTCTATTGTTCCCCGGTATATCCCAGGCTGCGGTCAGTGCCTGGCACATAGTAGATGCTCAATAGATATTTATCGATTGGAGGAACACAGCCCCACTCAACCCCAGGTTCCTACCGGGATACTCACGCACGGGGGTCTGGTTCACACCTGAAGCTGGAAACTGGGGGCTAATGTGAGCTCCTGTAGACGAGCCCCTAGTCCCAGACACTGTCTCTGCCATTAGCAGCACACAAAGCACACTCAGTGCCCAGCGCAGATTCAGCCTTCTGCCGGCCATCTGCCCCCACGGGGCCTGGCCCTTACTCTCCACTTCCCTTTCTGCAGTAGCTCAGTTCCAAACAGCTGGCGGCTCCGGTGGCCTGCAANGTGGGAGTGAGAAAGGGGGACAACATTACTGAAAACCCATGGACACAGCAAAAGGGGCTGGAAGACTCTAGTTAAAGGAACTGCCTTCCTGCCTCCCGGCAGCAAAGTTAAGCTGANGNAGAAACCATGNTCTGCACAGTCTCGCTGGGAGGGGCCCNNNNCCCTGCCCATTCAGCGTGCAGATCAGANNNCGAANTNCNNCAGNNNNNTATNNNNNTNTCGATACGTCGANNCGANGGGGGNNNNNNNCCCAGCTTTTNNNNNNNTNANNGANGNNNNNNNNNCNNNNNGTANCANGNNNCANNANNCNNNNTNNN

NCLX KO#33_2

NNNNNNNNNNATAGGGCGNTTGGAGCTCCCGCGGTGCGGCCGCTCTAGAACTAGTGGATCCCCCGGGCTGCAGCGACCAATGTGGAATTCGCCCTTCTGATCTGCACGCTGAATGGGCAGGGCCTTGGGCCCCTCCAGCGAGACTGTGCAGAACATGGTTTCTCCGTCAGCTTAACTTTGCTGCCGGGAGGCAGGAAGGCAGTTCCTTAACTAGAGTCTTCCAGCCCCTTTTGCTGTGTCCATGGGTTTTCAGTAATGTTGTCCCCCTTTCTCACTCCCACCTTGCAGGCCACCGGAGCCGCCAGCTGTTTGGAACTGAGCTACTGCAGAAAGGGAAGTGGAGAGTAAGGGCCAGGCCCCGTGGGGGCAGATGGCCGGCAGAAGGCTGAATCTGCGCTGGGCACTGAGTGTGCTTTGTGTGCTGCTAATGGCGGAGACAGTGTCTGGGACTAGGGGCTCGTCTACAGGAGCTCACATTAGCCCCCAGTTTCCAGCTTCAGGTGTGAACCAGACCCCCGTGAGACGTGAGTATCCCGGTAGGAACCTGGGGTTGAGTGGGGCTGTGTTCCTCCAATCGATAAATATCTATTGAGCATCTACTATGTGCCAGGCACTGACCGCAGCCTGGGATATACCGGGGAACAATAGAGACAAAATTCACTACTCTCAAGAAGCTGACAGTCTCCTGAACAGACAGATCAAGAAGTAAACTTATACACTTTGGGAGGCCAAGGCAGGAGGATCACTTGAGGCCAGGAGCTGGAGACCAGCCTGGCCATCGTGTCAAAACCCCGTATCTACTAAAAATACAAAAATTAGCTGGGCATGGTGGCAGGTGCCTGTAATCATCAGAACCCTGAGGCAGGAGAATTGCTTGAAACCGTGAGGTGGATGTTGCAGTGAGCCAGGATTGTACCACTGCACTCCAGCCTGGGCGGCGAAGCAAGACTCTGTCTCAACAACAAAAAAAAAAGANAAGTAAGCATATACATTTTCNNNNNNNATGTGNCTAAGTAAGNNNNNNNCNNCANNNNNATCAANGCTTATCGATACGTCGAACNNNAAGGNNNNNNNNCCAGNTTTNNNNNTANNNANNNNNNCGCGCTNGGNNNNN

NCLX KO #37_1

NNNNNNNNNNNNNNNNNNNNNGNCGAATTGGAGCTCCCGCGGTGCGGCCGCTCTAGAACT
AGTGGATCCCCCGGGCTGCNGNGNNNAATGTGGAATTCGCCCTTTCCAGCGAGACTGTGC
AGAACATGGTTTCTCCGTCAGCTTAACTTTGCTGCCGGGAGGCAGGAAGGCAGTTCCTTA
ACTGGAGTCTTCCAGCCCCTTTTGCTGTGTCCATGGGTTTTCAGTAATGTTGTCCCCCTT
TCTCACTCCCACCTTGCAGGCCACCGGAGCCGCCAGCTGTTTGGAACTGAGCTACTGCAG
AAAGGGAAGTGGAGAGTAAGGGCCAGGCCCCGTGGGGGCAGATGGCCGGCGAAGGCTGAA
TCTGCGCTGGGCACTGAGTGTGCTTTGTGTGCTGCTAATGGCGGAGACAGTGTCTGGGAC
TAGGGGCTCGTCTACAGGAGCTCACATTAGCCCCCAGTTTCCAGCTTCAGGTGTGAACCA
GACCCCCGTGGACGTGAGTATCCCGGTAGGAACCTGGGGTTGAGTGGGGCTGTGTTCCTC
CAATCGATAAATATCTATTGAGCATCTACTATGTGCCAGGCACTGACCGCAGCCTGGGAT
ATACCGGGGAACAATAGAGACAAAATTCACTACTCTCAAGAAGCTGACAGTCTCCTGAAC
AGACAGATCAAGAAGTAAACTTATACACTTTTGGGAGGCCAAGGCAGGAGGATCACTTGA
GGCCAGGAGCTGGANACCANCCTGGCCATCGTGTCAAAACCCCGTATCTACTAAAAATAC
AAAAATTANCTGGGCATGGTGGNAGGTGCCTGTAAATCATCAGAACCCTGAGGCAGGANA
ATTGCTTGAAACCGTGAGGTGNNTGTTGCANTGAGCCANGATTGTACCACCTGCACTCCA
NCCTGGGCGGNNAANCAAGACTCTGTCTCAACAACAAGGGCNGAATTCCACAGTGGATAT
CNNNCNNATCGATANCGTCNANCTCGAGGGGGGNCCCGGTACCCNNCNNTTGNTCCCTTT
AAGNGAAGGNTAATTGNGCGCTTGGNNNNANNNNNGGTCCATAGCTGNTTCNNNNNNN

NCLX KO #37_2

NNNNNNNNNNNNNNNNTNNNGGCNATTGGAGCTCCCGCGGTGCGGCCGCTCTAGAACTAG
TGGATCCCCCGGGCTGCAGNGNNNAATGTGGAATTCGCCCTTCAGCGAGACTGTGCAGAA
CATGGTTTCTCCGTCAGCTTAACTTTGCTGCCGGGAGGCAGGAAGGCAGTTCCTTAACTA
GAGTCTTCCAGCCCCTTTTGCTGTGTCCATGGGTTTTCAGTAATGTTGTCCCCCTTTCTC
ACTCCCACCTTGCAGGCCACCGGAGCCGCCAGCTGTTTGGAACTGAGCTACTGCAGAAAG
GGAAGTGGAGAGTAAGGGCCAGGCCCCGTGGGGGCAGATGGCCGGCAGTAGACGTGAGTA
TCCCGGTAGGAACCTGGGGTTGAGTGGGGCTGTGTTCCTCCAATCGATAAATATCTATTG
AGCATCTACTATGTGCCAGGCACTGACCGCAGCCTGGGATANNCCGGGGAACAATAGAGA
CAAAATTCACTACTCTCAAGAAGCTGACAGTCTCCTGAACAGACAGATCAATAAGTAAAC
TTATACACTTTGGGAGGTCAAGGCAGGAGGATCACTTGAGGCCAGGAGCTGGANACCANC
CTGGCCATCGTGTCACAACCTCGTATCTACTAAAAATACAAAANTTAGCTGGGCATGGNG
GCAGGTGCCTGTAATCATCAGAACCCTGANCAGGANAATTGCTTGAAACCGTGAGGTGNA
TGTTGCANNGAACCAGGATTGTACCANTGCACTCCATTCTGNNCGNGCCAANCANNANTC
TGTCTCNNNNNCANCGGCGAATTCCNCAGTGGATATCANCNTATCNATACCGNCGACCTC
NANGNGGGGTCCGGTACCANCTTTTGTTCNCTTTANTGAGGTNAATTGCGCGCTNGNCNN
ANANGGTCATANCTGTTTCCTGNNNGAAATNNTNNCCNCTNNNNTCCACNCANNATACNA
NNCNNNNNNNNAAGTNGTAAGCCNGNGNGNCNAATGATAGANCTANCTCCCANTNCANNG
NGNTGCNNNCNATNNNNNTTTCNAGTNCAGGAAANNNGNCGATGNCNNNNTGTNTN

NCLX KO #59_1

NNNNNNNNNNNNNNNNNNTNNNNNGAATTGGAGCTCCCGCGGTGCGGCCGCTCTAGAACT
AGTGGATCCCCCGGGCTGCNGCGACCAATGTGGAATTCGCCCTTTCCAGCGAGACTGTGC
AGAACATGGTTTCTCCGTCAGCTTAACTTTGCTGCCGGGAGGCAGGAAGGCAGTTCCTTA
ACTGGAGTCTTCCAGCCCCTTTTGCTGTGTCCATGGGTTTTCAGTAATGTTGTCCCCCTT
TCTCACTCCCACCTTGCAGGCCACCGGAGCCGCCAGCTGTTTGGAACTGAGCTACTGCAG
AAAGGGAAGTGGAGAGTAAGGGCCAGGCCCCGTGGGGGCAGATGGCCGGCAGTATCCCGG
TAGGAACCTGGGGTTGAGTGGGGCTGTGTTCCTCCAATCGATAAATATCTATTGAGCATC
TACTATGTGCCAGGCACTGACCGCAGCCTGGGATATACCGGGGAACAATAGAGACAAAAT
TCACTACTCNCAAGAAGCTGACAGTCTCCTGAACANACAGATCAAGAAGTAAACTTATNC
NCTTTGGGAGGCCAAGGCAGGAGGATCACTTGAGGCCAGGAGCTGGAGACCAGCCTGGCC
ATCGTGTCAAAACCCCGTATCTACTAAAAATACAAAAATTAGCTGGGCATGGTGGCAGGT
GCCTGTAATCATCAGAACCCTGAGGCAGGANAATTGCTTGAAACCNTGAGGGTGGATGTT
GCANTGAGCCAGGATTGTACCACTGCACTCCANCCTGNGNGGCGAANNANACTCTGTCTC
AACAACNNNGGNNANTTCNACNGNGGATATCAAGCTTATCGATACCGTCTACCTCTAGGG
GGGGNCCGGNACCCAGCTTTTGNTCCCTTTTNNTGATGNTTANTTGNNCGCTTGGNNNAT
CNTGGTCNTANCTGTNTTCCTGTGTNAATTGNTATCCNNNNNCAATTCACACAACNTACG
ANNCGNGANCATAAAGNGAAANNNNGGGGNNGCTAATGANTGANCTAANTCNCATTANNN
GNNTNGNGCNCNCTGCCCNCNNNCAANCNGNAANCTTNTNTNNNNANTTNNNTNNNTGAA
NTCGNNNACCCNNCNGNGANNN

NCLX KO #59_2

NNNNNNNNNNNNNNNNNNNGGCGANTTGGAGCTCCCGCGGTGCGGCCGCTCTAGAACTAG
TGGATCCCCCGGGCTGCAGCGACCAATGTGGAATTCGCCCTTTCCAGCGAGACTGTGCAG
AACATGGTTTCTCCGTCAGCTTAACTTTGCTGCCGGGAGGCAGGAAGGCAGTTCCTTAAC
TAGAGTCTTCCAGCCCCTTTTGCTGTGTCCATGGGTTTTCAGTAATGTTGTCCCCCTTTC
TCACTCCCACCTTGCAGGCCACCGGAGCCGCCAGCTGTTTGGAACTGAGCTACTGCAGAA
AGGGAAGTGGAGAGTAAGGGCCAGGCCCCGTGGGGGCAGATGGCCGGCACACGGGGGTCT
GGTTCACACCTGAAGCTGGAAACTGGGGGCTAATGTGAGCTCCTGTAGACGAGCCCCTAG
TCCCAGACACTGTCTCCGCCATTAGCAGCACACAAAGCACACTCAGTGCCCAGCGCAGAT
TCAGCCTTCGTAGACGTGAGTATCCCGGTAGGAACCTGGGGTTGAGTGGGGCTGTGTTCC
TCCAATCGATAAATATCTATTGAGCATCTACTATGTGCCAGGCACTGACCGCAGCCTGGG
ATATACCGGGGAACAATAGAGACAAAATTCACTACTCTCAAGAAGCTGACAGTCTCCTGA
ACAGACAGATCAAGAAGTAAACTTATACACTTTGGGAGGCCAAGGCAGGAGGATCACTTG
AGGCCAGGAGCTGGAGACCAGCCTGGCCATCGTGTCAAAACCCCGTATCTACTAAAAATA
CAAAAATTAGCTGGGCATGGTGGCAGGTGCCTGTAATCATCAGAACCCTGAGGCAGGANA
ATTGCTTGAAACCGTGAGGTGNATGTTGCAGTGAGCCAGGATTGTACCACTGCNCTCCAG
CCTGNCGGCGAAGCAAGACTCTGTCTCAACAACNAGGGGCGAATTCCACAGTGGATATCA
AGCTNTCGATACCGNCGACCTCNAAGGGGGNGGCCCGGTACCCAGCTTTGGTTCCCTTNN
GNGAGGGNNNNTNGNNCGCTTGGNGNNNTCATGGNACNNNANCNNGTTTCNNNNNN

**Sequencing of DLD1 NCLX KO Clones**

NCLXKO #06_1

NNNNNNNTNNNNNAGGGCGATTGGAGCTCCCGCGGTGCGGCCGCTCTAGAACTAGTGGATCCCCCGGGCTGCAGCGACCA
ATGTGGAATTCGCCCTTCTGATCTGCACGCTGAATGGGCAGGGCCTTGGGCCCCTCCAGCGAGACTGTGCAGAACATGGT
TTCTCCGTCAGCTTAACTTTGCTGCCGGGAGGCAGGAAGGCAGTTCCTTAACTAGAGTCTTCCAGCCCCTTTTGCTGTGT
CCATGGGTTTTCAGTAATGTTGTCCCCCTTTCTCACTCCCACCTTGCAGGCCACCGGAGCCGCCAGCTGTTTGGAACTGA
GCTACTGCAGAAAGGGAAGTGGAGAGTAAGGGCCAGGCCCCGTGGGGGCAGATGGCCGGCAGAAGGCTGAATCTGCGCTG
GGCACTGAGTGTGCTTTGTGTGCTGCTAATGGCGGAGACAGTGTCTGGGACTAGGGGCTCGTCTACAGGAGCTCACATTA
GCCCCCAGTTTCCAGCTTCAGGTGTGAACCAGACCCCCGTGGTAGACGTGAGTATCCCGGACTGCTGGTGTGGGTCCTGG
CAGGCGCCCTGGGGCTCAGCCTCGTCTTCTCCCTGGTCTCAGTCCCATTGCAGTGCTTCCAGCTCAGCAGAGTCTATGGC
TTCTGCCTGCTCCTCTTCTACCTGAACTTCCTTGTCGTGGCCCTCCTCACTGAATTTGGAGTGATTCACCTGAAAAGCAT
GTGACTGAAGCCGCTTAGTGCTGTGGCCTCACTGCAGGCAGGAGCCCCGCCCCTCCTGCCGGGGGANGCCCAGGGACCGG
AGCATTTCTGCAAGGCCCTTGTGGGCACGAGAGTGCGGCCCTTGCTGCTGGAGATCTGNNTCACTGCTGTGAGCTGGGAN
AACTGCTGTGTACCTCANGGCGAATTCCACAGTGGNTATCAAGCTTATCGATACCGTCGACCTCGAGGGGGGGCCCNGGT
ACCCAGCTTNN

NCLXKO #06_2

NNNNNNNNNTNNNNNNGGGNNNNNGGAGCTCCCGCGGTGCGGCCGCTCTAGAACTAGTGGATCCCCCGGGCTGCAGCGAC
CAATGTGGAATTCGCCCTTCTGATCTGCACGCTGAATGGGCAGGGCCTTGGGCCCCTCCAGCGAGACTGTGCAGAACATG
GTTTCTCCGTCAGCTTAACTTTGCTGCCGGGAGGCAGGAAGGCAGTTCCTTAACTAGAGTCTTCCAGCCCCTTTTGCTGT
GTCCATGGGTTTTCAGTAATGTTGTCCCCCTTTCTCACTCCCACCTTGCAGGCCACCGGAGCCGCCAGCTGTTTGGAACT
GAGCTACTGCAGAAAGGGAAGTGGAGAGTAAGGGCCAGGCCCCGTGGGGGCAGATGGCCGGCAGAAGGCTGAATCTGCGC
TGGGCACTGAGTGTGCTTTGTGTGCTGCTAATGGCGGAGACAGTGTCTGGGACTAGGGGCTCGTCTACAGGAGCTCACAT
TAGCCCCCAGTTTCCAGCTTCAGGTGTGAACCAGACCCCCGTGGTAGACGTGAGTATCCCGGACTGCTGGTGTGGGTCCT
GGCAGGCGCCCTGGGGCTCAGCCTCGTCTTCTCCCTGGTCTCAGTCCCATTGCAGTGCTTCCAGCTCAGCAGAGTCTATG
GCTTCTGCCTGCTCCTCTTCTACCTGAACTTCCTTGTCGTGGCCCTCCTCACTGAATTTGGAGTGATTCACCTGAAAAGC
ATGTGACTGAAGCCGCTTAGTGCTGTGGCCTCACTGCAGGCAGGAGCCCCGCCCCTCCTGCCGGGGGANGCCCAGGGACC
GGAGCATTTCTGCAAGGCCCTTGTGGGCACGAGAGTGCGGCCCTTGCTGCTGGAGATCTGAGGTCACTGCTGTGAGCTGG
GAGAACTGCTGTGTACCTCANNGGCGAATTCCACAGTGGNTATCAAGCTTATCGATACCGTCGACCTCGAGGGGGGGCCC
GGTACCCAGCNN

NCLXKO #06_3

NNNNNNNNNNNNNNNAGGGCGATTGGAGCTCCCGCGGTGCGGCCGCTCTAGAACTAGTGGATCCCCCGGGCTGCAGCGAC
CAATGTGGAATTCGCCCTTGAGGTACACAGCAGTTCTCCCAGCTCACAGCAGTGACCTCAGATCTCCAGCAGCAAGGGCC
GCACTCTCGTGCCCACAAGGGCCTTGCAGAAATGCTCCGGTCCCTGGGCCTCCCCCGGCAGGAGGGGCGGGGCTCCTGCC
TGCAGTGAGGCCACAGCACTAAGCGGCTTCAGTCACATGCTTTTCAGGTGAATCACTCCAAATTCAGTGAGGAGGGCCAC
GACAAGGAAGTTCAGGTAGAAGAGGAGCAGGCAGAAGCCATAGACTCTGCTGAGCTGGAAGCACTGCAATGGGACTGAGA
CCAGGGAGAAGACGAGGCTGAGCCCCAGGGCGCCTGCCAGGACCCACACCAGCAGTCCGGGATACTCACGTCTACCACGG
GGGTCTGGTTCACACCTGAAGCTGGAAACTGGGGGCTAATGTGAGCTCCTGTAGACGAGCCCCTAGTCCCAGACACTGTC
TCCGCCATTAGCAGCACACAAAGCACACTCAGTGCCCAGCGCAGATTCAGCCTTCTGCCGGCCATCTGCCCCCACGGGGC
CTGGCCCTTACTCTCCACTTCCCTTTCTGCAGTAGCTCAGTTCCAAACAGCTGGCGGCTCCGGTGGCCTGCAAGGTGGGA
GTGAGAAAGGGGGACAACATTACTGAAAACCCATGGACACAGCAAAAGGGGCTGGAAGACTCTAGTTAAGGAACTGCCTT
CCTGCCTCCCGGCAGCAAAGTTAAGCTGACGGAGAAACCATGTTCTGCACAGTCTCGCTGGAGGGGCCCNAAGGCCCTGC
CCATTCAGCGTGCAGATCAGANGGCGAATTCCACAGTGGNTATCAAGCTTATCGATACCGTCGACCTCNAGGGGGGGCCC
NGGTACCNAGCTTTTGNTCCCTTTANTGAGGGTTAATTGCGNNCTTGGCGTAATCATGGGNN

NCLXKO #06_4

NNNNNNNNTNNNNNNGGGCGAATTGGAGCTCCCGCGGTGCGGCCGCTCTAGAACTAGTGGATCCCCCGGGCTGCAGCGAC
CAATGTGGAATTCGCCCTTCTGATCTGCACGCTGAATGGGCAGGGCCTTGGGCCCCTCCAGCGAGACTGTGCAGAACATG
GTTTCTCCGTCAGCTTAACTTTGCTGCCGGGAGGCAGGAAGGCAGTTCCTTAACTAGAGTCTTCCAGCCCCTTTTGCTGT
GTCCATGGGTTTTCAGTAATGTTGTCCCCCTTTCTCACTCCCACCTTGCAGGCCACCGGAGCCGCCAGCTGTTTGGAACT
GAGCTACTGCAGAAAGGGAAGTGGAGAGTAAGGGCCAGGCCCCGTGGGGGCAGATGGCCGGCAGAAGGCTGAATCTGCGC
TGGGCACTGAGTGTGCTTTGTGTGCTGCTAATGGCGGAGACAGTGTCTGGGACTAGGGGCTCGTCTACAGGAGCTCACAT
TAGCCCCCAGTTTCCAGCTTCAGGTGTGAACCAGACCCCCGTGGTAGACGTGAGTATCCCGGACTGCTGGTGTGGGTCCT
GGCAGGCGCCCTGGGGCTCAGCCTCGTCTTCTCCCTGGTCTCAGTCCCATTGCAGTGCTTCCAGCTCAGCAGAGTCTATG
GCTTCTGCCTGCTCCTCTTCTACCTGAACTTCCTTGTCGTGGCCCTCCTCACTGAATTTGGAGTGATTCACCTGAAAAGC
ATGTGACTGAAGCCGCTTAGTGCTGTGGCCTCACTGCAGGCAGGAGCCCCGCCCCTCCTGCCGGGGGAGGCCCAGGGACC
GGAGCATTTCTGCAAGGCCCTTGTGGGCACGAGAGTGCGGCCCTTGCTGCTGGAGATCTGAGGTCACTGCTGTGAGCTGG
GAGAACTGCTGTGTACCTCAAGGGCGAATTCCACAGTGGNTATCAAGCTTATCGATACCGTCGACCTCNNNGGGGGCCCG
GTACCCAGCTTTTGTTCCCTTTANTGAGGGTTAATTGCGNGCTTNGNGTAATCATGGNN

NCLXKO #06_5

NNNNNNNNTNNNNNAGGGCGATTGGAGCTCCCGCGGTGCGGCCGCTCTAGAACTAGTGGATCCCCCGGGCTGCAGCGACC
AATGTGGAATTCGCCCTTGAGGTACACAGCAGTTCTCCCAGCTCACAGCAGTGACCTCAGATCTCCAGCAGCAAGGGCCG
CACTCTCGTGCCCACAAGGGCCTTGCAGAAATGCTCCGGTCCCTGGGCCTCCCCCGGCAGGAGGGGCGGGGCTCCTGCCT
GCAGTGAGGCCACAGCACTAAGCGGCTTCAGTCACATGCTTTTCAGGTGAATCACTCCAAATTCAGTGAGGAGGGCCACG
ACAAGGAAGTTCAGGTAGAAGAGGAGCAGGCAGAAGCCATAGACTCTGCTGAGCTGGAAGCACTGCAATGGGACTGAGAC
CAGGGAGAAGACGAGGCTGAGCCCCAGGGCGCCTGCCAGGACCCACACCAGCAGTCCGGGATACTCACGTCTACCACGGG
GGTCTGGTTCACACCTGAAGCTGGAAACTGGGGGCTAATGTGAGCTCCTGTAGACGAGCCCCTAGTCCCAGACACTGTCT
CCGCCATTAGCAGCACACAAAGCACACTCAGTGCCCAGCGCAGATTCAGCCTTCTGCCGGCCATCTGCCCCCACGGGGCC
TGGCCCTTACTCTCCACTTCCCTTTCTGCAGTAGCTCAGTTCCAAACAGCTGGCGGCTCCGGTGGCCTGCAAGGTGGGAG
TGAGAAAGGGGGACAACATTACTGAAAACCCATGGACACAGCAAAANNNNTGGAAGACTCTAGTTAAGGAACTGCCTTCC
TGCCTCCCGGCAGCAAAGTTAAGCTGACGGAGAAACCATGTTCTGCACAGTCTCGCTGGNGGGGCCCAAAGGCCCTGCCC
ATTCAGCGTGCAGATCAGANGGCGAANTTCCACAGNGGNNATCAAGCTTATCGATACCGTCGACCTCGAGGGGGGGCCCG
GTACCCAGCN

NCLXKO #24_1

NNNNNNNNNTNNNNTAGGGCGATTGGAGCTCCCGCGGTGCGGCCGCTCTAGAACTAGTGGATCCCCCGGGCTGCAGCGAC
CAATGTGGAATTCGCCCTTCTGATCTGCACGCTGAATGGGCAGGGCCTTGGGCCCCTCCAGCGAGACTGTGCAGAACATG
GTTTCTCCGTCAGCTTAACTTTGCTGCCGGGAGGCAGGAAGGCAGTTCCTTAACTAGAGTCTTCCAGCCCCTTTTGCTGT
GTCCATGGGTTTTCAGTAATGTTGTCCCCCTTTCTCACTCCCACCTTGCAGGCCACCGGAGCCGCCAGCTGTTTGGAACT
GAGCTACTGCAGAAAGGGAAGTGGAGAGTAAGGGCCAGGCCCCGTGGGGGCAGATGGCCGGCAGAAGGCTGAATCTGCGC
TGGGCACTGAGTGTGCTTTGTGTGCTGCTAATGGCGGAGACAGTGTCTGGGACTAGGGGCTCGTCTACAGGAGCTCACAT
TAGCCCCCAGTTTCCAGCTTCAGGTGTGAACCAGACCCCCGTGGTAGACGTGAGTATCCCGACTGCTGGTGTGGGTCCTG
GCAGGCGCCCTGGGGCTCAGCCTCGTCTTCTCCCTGGTCTCAGTCCCATTGCAGTGCTTCCAGCTCAGCAGAGTCTATGG
CTTCTGCCTGCTCCTCTTCTACCTGAACTTCCTTGTCGTGGCCCTCCTCACTGAATTTGGAGTGATTCACCTGAAAAGCA
TGTGACTGAAGCCGCTTAGTGCTGTGGCCTCACTGCAGGCAGGAGCCCCGCCCCTCCTGCCGGGGGAGGCCCAGGGACCG
GAGCATTTCTGCAAGGCCCTTGTGGGCACGANAGTGCGGCCCTTGCTGCTGGAGATCTGANTCACTGCTGTGAGCTGGGA
GAACTGCTGTGTACCTCAAGNNGAATTCCACAGNGGNTATCAAGCTNATCGATACCN

NCLXKO #24_2

NNNNNNNTNNNNTAGGGCGATTGGAGCTCCCGCGGTGCGGCCGCTCTAGAACTAGTGGATCCCCCGGGCTGCAGCGACCA
ATGTGGAATTCGCCCTTCTGATCTGCACGCTGAATGGGCAGGGCCTTGGGCCCCTCCAGCGAGACTGTGCAGAACATGGT
TTCTCCGTCAGCTTAACTTTGCTGCCGGGAGGCAGGAAGGCAGTTCCTTAACTAGAGTCTTCCAGCCCCTTTTGCTGTGT
CCATGGGTTTTCAGTAATGTTGTCCCCCTTTCTCACTCCCACCTTGCAGGCCACCGGAGCCGCCAGCTGTTTGGAACTGA
GCTACTGCAGAAAGGGAAGTGGAGAGTAAGGGCCAGGCCCCGTGGGGGCAGATGGCCGGCAGAAGGCTGAATCTGCGCTG
GGCACTGAGTGTGCTTTGTGTGCTGCTAATGGCGGAGACAGTGTCTGGGACTAGGGGCTCGTCTACAGGAGCTCACATTA
GCCCCCAGTTTCCAGCTTCAGGTGTGAACCAGACCCCCGTGGTAGACGTGAGTATCCCGACTGCTGGTGTGGGTCCTGGC
AGGCGCCCTGGGGCTCAGCCTCGTCTTCTCCCTGGTCTCAGTCCCATTGCAGTGCTTCCAGCTCAGCAGAGTCTATGGCT
TCTGCCTGCTCCTCTTCTACCTGAACTTCCTTGTCGTGGCCCTCCTCACTGAATTTGGAGTGATTCACCTGAAAAGCATG
TGACTGAAGCCGCTTAGTGCTGTGGCCTCACTGCAGGCAGGAGCCCCGCCCCTCCTGCCGGGGGANGCCCAGGGACCGGA
GCATTTCTGCAAGGCCCTTGTGGGCACGAGAGTGCGGCCCTTGCTGCTGGAGATCTGAGGTCACTGCTGTGAGCTGGGAG
AACTGCTGTGTACCTCAANGGCGAATTCCACAGTGGNTATCAAGCTTATCGATACCGTCGACCTCGAGGGGGGGCCCGGT
NN

NCLXKO #24_3

NNNNNNNTNNNTAGGGCGATTGGAGCTCCCGCGGTGCGGCCGCTCTAGAACTAGTGGATCCCCCGGGCTGCAGCGACCAA
TGTGGAATTCGCCCTTCTGATCTGCACGCTGAATGGGCAGGGCCTTGGGCCCCTCCAGCGAGACTGTGCAGAACATGGTT
TCTCCGTCAGCTTAACTTTGCTGCCGGGAGGCAGGAAGGCAGTTCCTTAACTAGAGTCTTCCAGCCCCTTTTGCTGTGTC
CATGGGTTTTCAGTAATGTTGTCCCCCTTTCTCACTCCCACCTTGCAGGCCACCGGAGCCGCCAGCTGTTTGGAACTGAG
CTACTGCAGAAAGGGAAGTGGAGAGTAAGGGCCAGGCCCCGTGGGGGCAGATGGCCGGCAGAAGGCTGAATCTGCGCTGG
GCACTGAGTGTGCTTTGTGTGCTGCTAATGGCGGAGACAGTGTCTGGGACTAGGGGCTCGTCTACAGGAGCTCACATTAG
CCCCCAGTTTCCAGCTTCAGGTGTGAACCAGACCCCCGTGGTAGACGTGAGTATCCCGACTGCTGGTGTGGGTCCTGGCA
GGCGCCCTGGGGCTCAGCCTCGTCTTCTCCCTGGTCTCAGTCCCATTGCAGTGCTTCCAGCTCAGCAGAGTCTATGGCTT
CTGCCTGCTCCTCTTCTACCTGAACTTCCTTGTCGTGGCCCTCCTCACTGAATTTGGAGTGATTCACCTGAAAAGCATGT
GACTGAAGCCGCTTAGTGCTGTGGCCTCACTGCAGGCAGGAGCCCCGCCCCTCCTGCCGGGGGANGCCCAGGGACCGGAG
CATTTCTGCAAGCCCTTGTGGGCACGAGAGTGCGGCCCTTGCTGCTGGAGATCTGAGGTCACTGCTGTGAGCTGGGAGAA
CTGCTGTGTACCTCAAGGGCGAATTCCACAGTGGNTATCAAGCTTATCGATACCGTCGACCTCGAGGGGGGGCCCGGNAC
CNNNN

NCLXKO #24_4

NNNNNNNNTNNNTAGGGCGATTGGAGCTCCCGCGGTGCGGCCGCTCTAGAACTAGTGGATCCCCCGGGCTGCAGCGACCA
ATGTGGAATTCGCCCTTGAGGTACACAGCAGTTCTCCCAGCTCACAGCAGTGACCTCAGATCTCCAGCAGCAAGGGCCGC
ACTCTCGTGCCCACAAGGGCCTTGCAGAAATGCTCCGGTCCCTGGGCCTCCCCCGGCAGGAGGGGCGGGGCTCCTGCCTG
CAGTGAGGCCACAGCACTAAGCGGCTTCAGTCACATGCTTTTCAGGTGAATCACTCCAAATTCAGTGAGGAGGGCCACGA
CAAGGAAGTTCAGGTAGAAGAGGAGCAGGCAGAAGCCATAGACTCTGCTGAGCTGGAAGCACTGCAATGGGACTGAGACC
AGGGAGAAGACGAGGCTGAGCCCCAGGGCGCCTGCCAGGACCCACACCAGCAGTCGGGATACTCACGTCTACCACGGGGG
TCTGGTTCACACCTGAAGCTGGAAACTGGGGGCTAATGTGAGCTCCTGTAGACGAGCCCCTAGTCCCAGACACTGTCTCC
GCCATTAGCAGCACACAAAGCACACTCAGTGCCCAGCGCAGATTCAGCCTTCTGCCGGCCATCTGCCCCCACGGGGCCTG
GCCCTTACTCTCCACTTCCCTTTCTGCAGTAGCTCAGTTCCAAACAGCTGGCGGCTCCGGTGGCCTGCAAGGTGGGAGTG
AGAAAGGGGGACAACATTACTGAAAACCCATGGACACAGCAAAAGGGGCTGGAAGACTCTAGTTAAGGAACTGCCTTCCT
GCCTCCCGGCAGCAAAGTTAAGCTGACGGAGAAACCATGTTCTGCACAGTCTCGCTGGNGGGGCCCNAAGGCCCTGCNCA
TTCAGCGTGCAGATCAGANNNCGAATTCCACAGNGGNNATCAAGCTTATCGATACCGTCGACCTCGAGGGGGGGCNCGGT
ANNNCAN

NCLXKO #24_5

NNNNNNNNNNNTAGGGCGATTGGAGCTCCCGCGGTGCGGCCGCTCTAGAACTAGTGGATCCCCCGGGCTGCAGCGACCAA
TGTGGAATTCGCCCTTGAGGTACACAGCAGTTCTCCCAGCTCACAGCAGTGACCTCAGATCTCCAGCAGCAAGGGCCGCA
CTCTCGTGCCCACAAGGGCCTTGCAGAAATGCTCCGGTCCCTGGGCCTCCCCCGGCAGGAGGGGCGGGGCTCCTGCCTGC
AGTGAGGCCACAGCACTAAGCGGCTTCAGTCACATGCTTTTCAGGTGAATCACTCCAAATTCAGTGAGGAGGGCCACGAC
AAGGAAGTTCAGGTAGAAGAGGAGCAGGCAGAAGCCATAGACTCTGCTGAGCTGGAAGCACTGCAATGGGACTGAGACCA
GGGAGAAGACGAGGCTGAGCCCCAGGGCGCCTGCCAGGACCCACACCAGCAGTCGGGATACTCACGTCTACCACGGGGGT
CTGGTTCACACCTGAAGCTGGAAACTGGGGGCTAATGTGAGCTCCTGTAGACGAGCCCCTAGTCCCAGACACTGTCTCCG
CCATTAGCAGCACACAAAGCACACTCAGTGCCCAGCGCAGATTCAGCCTTCTGCCGGCCATCTGCCCCCACGGGGCCTGG
CCCTTACTCTCCACTTCCCTTTCTGCAGTAGCTCAGTTCCAAACAGCTGGCGGCTCCGGTGGCCTGCAAGGTGGGAGTGA
GAAAGGGGGACAACATTACTGAAAACCCATGGACACAGCAAAAGGGGCTGGAAGACTCTAGTTAAGGAACTGCCTTCCTG
CCTCCCGGCAGCAAAGTTAAGCTGACGGAGAAACCATGTTCTGCACAGTCTCGCTGGNGGGGCCCNAAGGCCCTGCCCAT
TCAGCGTGCAGATCAGAAAGGGCGAATTCCACAGTGGNTATCAAGCTTATCGATACCGTCGACCTCGAGGGGGGGCCCNG
GTACCCAGCTTTTGTTCCCTTTAGTGNNGGGTTAATTGCGCGCTTGGNGTAATC

NCLXKO #32_1

NNNNNNANTNNNNTAGGGCGANTGGAGCTCCCGCGGTGCGGCCGCTCTAGAACTAGTGGATCCCCCGGGCTGCAGCGACC

AATGTGGAATTCGCCCTTCTGATCTGCACGCTGAATGGGCAGGGCCTTGGGCCCCTCCAGCGAGACTGTGCAGAACATGG

TTTCTCCGTCAGCTTAACTTTGCTGCCGGGAGGCAGGAAGGCAGTTCCTTAACTAGAGTCTTCCAGCCCCTTTTGCTGTG

TCCATGGGTTTTCAGTAATGTTGTCCCCCTTTCTCACTCCCACCTTGCAGGCCACCGGAGCCGCCAGCTGTTTGGAACTG

AGCTACTGCAGAAAGGGAAGTGGAGAGTAAGGGCCAGGCCCCGTGGGGGCAGATGGCCGGCAGAAGGCTGAATCTGCGCT

GGGCACTGAGTGTGCTTTGTGTGCTGCTAATGGCGGAGACAGTGTCTGGGACTAGGGGCTCGTCTACAGGAGCTCACATT

AGCCCCCAGTTTCCAGCTTCAGGTGTGAACCAGACCCCCGTGGTAGACGTGAGTATCCCGGACTGCTGGTGTGGGTCCTG

GCAGGCGCCCTGGGGCTCAGCCTCGTCTTCTCCCTGGTCTCAGTCCCATTGCAGTGCTTCCAGCTCAGCAGAGTCTATGG

CTTCTGCCTGCTCCTCTTCTACCTGAACTTCCTTGTCGTGGCCCTCCTCACTGAATTTGGAGTGATTCACCTGAAAAGCA

TGTGACTGAAGCCGCTTAGTGCTGTGGCCTCACTGCAGGCAGGAGCCCCGCCCCTCCTGCCGGGGGANGCCCAGGGACCG

GAGCATTTCTGCAAGGCCCTTGTGGGCACGAGAGTGCGGCCCTTGCTGCTGGAGATCTGAGGTCACTGCTGTGAGCTGGG

ANAACTGCTGTGTACCTCANGGNGAATTCCACAGTGGNTATCAAGCTTATCGATACCGTCGACCTCNAGGGGGGGCCCGG

TACCCAGCTTTTGTTCCCN

NCLXKO #32_2

NNNNNNNTNNNNNNGGGCGATTGGAGCTCCCGCGGTGCGGCCGCTCTAGAACTAGTGGATCCCCCGGGCTGCAGCGACCA

ATGTGGAATTCGCCCTTCTGATCTGCACGCTGAATGGGCAGGGCCTTGGGCCCCTCCAGCGAGACTGTGCAGAACATGGT

TTCTCCGTCAGCTTAACTTTGCTGCCGGGAGGCAGGAAGGCAGTTCCTTAACTAGAGTCTTCCAGCCCCTTTTGCTGTGT

CCATGGGTTTTCAGTAATGTTGTCCCCCTTTCTCACTCCCACCTTGCAGGCCACCGGAGCCGCCAGCTGTTTGGAACTGA

GCTACTGCAGAAAGGGAAGTGGAGAGTAAGGGCCAGGCCCCGTGGGGGCAGATGGCCGGCAGAAGGCTGAATCTGCGCTG

GGCACTGAGTGTGCTTTGTGTGCTGCTAATGGCGGAGACAGTGTCTGGGACTAGGGGCTCGTCTACAGGAGCTCACATTA

GCCCCCAGTTTCCAGCTTCAGGTGTGAACCAGACCCCCGTGGTAGACGTGAGTATCCCGGACTGCTGGTGTGGGTCCTGG

CAGGCGCCCTGGGGCTCAGCCTCGTCTTCTCCCTGGTCTCAGTCCCATTGCAGTGCTTCCAGCTCAGCAGAGTCTATGGC

TTCTGCCTGCTCCTCTTCTACCTGAACTTCCTTGTCGTGGCCCTCCTCACTGAATTTGGAGTGATTCACCTGAAAAGCAT

GTGACTGAAGCCGCTTAGTGCTGTGGCCTCACTGCAGGCAGGAGCCCCGCCCCTCCTGCCGGGGGAGGCCCAGGGACCGG

AGCATTTCTGCAAGNCCTTGTGGGCACGAGAGTGCGGCCCTTGCTGCTGNNATCTGANTCACTGCTNTGAGCTNGGANNA

CTGCTGNNTACTCNNGGCNANNCNNNGNGNNATCAGCTNTCGATNCNNCNACNCNAGGGGGGGNNNNNCNNCTTNNNNNN

NNNNGNANNNCNCNNGNNNANCNNGNNNNCNNTNNNNNNNNNNNNNNCCNNNNNNNTCCNNNNNANNNNNNNNNNNNNNN

NNNNNNNNANNNNN

NCLXKO #32_3

NNNNNNNNTNNNNNAGGGCGATTGGAGCTCCCGCGGTGCGGCCGCTCTAGAACTAGTGGATCCCCCGGGCTGCAGCGACC

AATGTGGAATTCGCCCTTGGTACACAGCAGTTCTCCCAGCTCACAGCAGTGACCTCAGATCTCCAGCAGCAAGGGCCGCA

CTCTCGTGCCCACAAGGGCCTTGCAGAAATGCTCCGGTCCCTGGGCCTCCCCCGGCAGGAGGGGCGGGGCTCCTGCCTGC

AGTGAGGCCACAGCACTAAGCGGCTTCAGTCACATGCTTTTCAGGTGAATCACTCCAAATTCAGTGAGGAGGGCCACGAC

AAGGAAGTTCAGGTAGAAGAGGAGCAGGCAGAAGCCATAGACTCTGCTGAGCTGGAAGCACTGCAATGGGACTGAGACCA

GGGAGAAGACGAGGCTGAGCCCCAGGGCGCCTGCCAGGACCCACACCAGCAGTCCGGGATACTCACGTCTACCACGGGGG

TCTGGTTCACACCTGAAGCTGGAAACTGGGGGCTAATGTGAGCTCCTGTAGACGAGCCCCTAGTCCCAGACACTGTCTCC

GCCATTAGCAGCACACAAAGCACACTCAGTGCCCAGCGCAGATTCAGCCTTCTGCCGGCCATCTGCCCCCACGGGGCCTG

GCCCTTACTCTCCACTTCCCTTTCTGCAGTAGCTCAGTTCCAAACAGCTGGCGGCTCCGGTGGCCTGCAAGGTGGGAGTG

AGNNNGGGGACAACATTACTGAAAACCCATGGACACAGCAAAAGGGGCTGGAAGACTCTAGTTAAGGAACTGCCTTCCTG

CCTCCCGGCAGCAAAGTTAAGCTGACGGAGAAACCATGTTCTGCACAGTCTCGCTGGAGGGGCCCAAGGCCCTGCCCATT

CAGCGTGCAGATCAGANNNCGAATTCCACAGTGGNNATCAAGCTTATCGATACCGTCGACCTCNAGGGGGGGCCCGGTAC

CCAGCTTTTGTTCCCTTTANTGANGGNNNANTTGCNN

NCLXKO #32_4

NNNNNNNTNNNNTAGGGCGATTGGAGCTCCCGCGGTGCGGCCGCTCTAGAACTAGTGGATCCCCCGGGCTGCAGCGACCA
ATGTGGAATTCGCCCTTGAGGTACACAGCAGTTCTCCCAGCTCACAGCAGTGACCTCAGATCTCCAGCAGCAAGGGCCGC
ACTCTCGTGCCCACAAGGGCCTTGCAGAAATGCTCCGGTCCCTGGGCCTCCCCCGGCAGGAGGGGCGGGGCTCCTGCCTG
CAGTGAGGCCACAGCACTAAGCGGCTTCAGTCACATGCTTTTCAGGTGAATCACTCCAAATTCAGTGAGGAGGGCCACGA
CAAGGAAGTTCAGGTAGAAGAGGAGCAGGCAGAAGCCATAGACTCTGCTGAGCTGGAAGCACTGCAATGGGACTGAGACC
AGGGAGAAGACGAGGCTGAGCCCCAGGGCGCCTGCCAGGACCCACACCAGCAGTCCGGGATACTCACGTCTACCACGGGG
GTCTGGTTCACACCTGAAGCTGGAAACTGGGGGCTAATGTGAGCTCCTGTAGACGAGCCCCTAGTCCCAGACACTGTCTC
CGCCATTAGCAGCACACAAAGCACACTCAGTGCCCAGCGCAGATTCAGCCTTCTGCCGGCCATCTGCCCCCACGGGGCCT
GGCCCTTACTCTCCACTTCCCTTTCTGCAGTAGCTCAGTTCCAAACAGCTGGCGGCTCCGGTGGCCTGCAAGGTGGGAGT
GAGAAAGGGGGACAACATTACTGAAAACCCATGGACACAGCAAAAGGGGCTGGAAGACTCTAGTTAANNAACTGCCTTCC
TGCCTCCCGGCAGCAAAGTTAAGCTGACGGAGAAACCATGTTCTGCACAGTCTCGCTGGAGGGGCCCNAAGGCCCTGCCN
ATTCAGCGTGCAGATCAGAANGGCGAANTTCCACAGTGGNTATCAAGCTTATCGATACCGTCGACCTCNAGGGGGGGCCC
GGTACCCAGCTTTTGTTCCCTTTANTGAGGGTTAATTGCGCNCTTNGNGTAATCAN

NCLXKO #32_5

NNNNNNNNNNNNNAGGGCGATTGGAGCTCCCGCGGTGCGGCCGCTCTAGAACTAGTGGATCCCCCGGGCTGCAGCGACCA
ATGTGGAATTCGCCCTTGAGGTACACAGCAGTTCTCCCAGCTCACAGCAGTGACCTCAGATCTCCAGCAGCAAGGGCCGC
ACTCTCGTGCCCACAAGGGCCTTGCAGAAATGCTCCGGTCCCTGGGCCTCCCCCGGCAGGAGGGGCGGGGCTCCTGCCTG
CAGTGAGGCCACAGCACTAAGCGGCTTCAGTCACATGCTTTTCAGGTGAATCACTCCAAATTCAGTGAGGAGGGCCACGA
CAAGGAAGTTCAGGTAGAAGAGGAGCAGGCAGAAGCCATAGACTCTGCTGAGCTGGAAGCACTGCAATGGGACTGAGACC
AGGGAGAAGACGAGGCTGGTCTGGTTCACACCTGAAGCTGGAAACTGGGGGCTAATGTGAGCTCCTGTAGACGAGCCCCT
AGTCCCAGACACTGTCTCCGCCATTAGCAGCACACAAAGCACACTCAGTGCCCAGCGCAGATTCAGCCTTCTGCCGGCCA
TCTGCCCCCACGGGGCCTGGCCCTTACTCTCCACTTCCCTTTCTGCAGTAGCTCAGTTCCAAACAGCTGGCGGCTCCGGT
GGCCTGCAAGGTGGGAGTGAGAAAGGGGGACAACATTACTGAAAACCCATGGACACAGCAAAAGGGGCTGGAAGACTCTA
GTTAAGGAACTGCCTTCCTGCCTCCCGGCAGCAAAGTTAAGCTGACGGAGAAACCATGTTCTGCACAGTCTCGCTGGAGG
GGCCCNAAGGCCCTGCCCATTCAGCGTGCAGATCAGAAAGGGCGAATTCCACAGTGGATATCAAGCTTATCGATACCGTC
GACCTCGAGGGGGGGCCCGGTACCCAGCTTTTGTTCCCTTTANTGAGGGTTAATTGCGCGCTTGGNGTAATCATGGGTCA
TAGCNNNN
